# The LINC01315-encoded small protein YAPer-ORF competes with PRP4k to hijack YAP signaling to aberrantly promote cell growth

**DOI:** 10.1038/s41418-025-01449-z

**Published:** 2025-02-17

**Authors:** Zhu Xie, Chao Li, Rui Huang, Bo Wu, Qian Huang, Zhe Zhang, Tongjin Zhao, Lingqian Wu, Chengtao Li, Jianfeng Shen, Hongyan Wang

**Affiliations:** 1 Obstetrics and Gynecology Hospital, State Key Laboratory of Genetic Engineering, Shanghai Key Laboratory of Metabolic Remodeling and Health, Institute of Metabolism and Integrative Biology, Fudan University, Shanghai, 200438 China; 2 Department of Ophthalmology, Ninth People’s Hospital, Shanghai JiaoTong University School of Medicine, Institute of Translational Medicine, National Facility for Translational Medicine, Shanghai JiaoTong University, Shanghai, 200240 China; 3 Prenatal Diagnosis Center of Shenzhen Maternity & Child Healthcare Hospital, Shenzhen, 518028 China; 4 Cancer Center, Shanghai General Hospital, Shanghai Jiao Tong University School of Medicine, Shanghai, 200081 China; 5 Shanghai Key Laboratory of Metabolic Remodeling and Health, Institute of Metabolism and Integrative Biology, Fudan University, Shanghai, 200438 China; 6 Center for Medical Genetics, Hunan Key Laboratory of Medical Genetics & Hunan Key Laboratory of Animal Models for Human Diseases, School of Life Sciences, Central South University, Changsha, 410078 China; 7 Shanghai Medical College, Fudan University, Shanghai, 200032 China; 8 Shenzhen Maternal and Child Health Hospital, Shenzhen, 518000 China

**Keywords:** Epigenetics, RNA

## Abstract

The dysregulation of YAP activity is implicated in abnormal organ size and the pathogenesis of diverse diseases, including cancer. However, the functional regulation of YAP activity by lncRNA-encoded peptides remains elusive. In this study, we report the identification of a small protein (93 aa) encoded by the lncRNA LINC01315. This small protein, termed YAPer-ORF, preferentially interacted with GNAQ/11 mutants to augment YAP activity. Mechanistically, YAPer-ORF was located in the nucleus and competed with YAP to bind the nuclear kinase PRP4K to hinder YAP phosphorylation. This decreased phosphorylation of YAP by YAPer-ORF promoted YAP retention in the nucleus and facilitated the expression of downstream target genes such as *CCND1*. In both cancerous and noncancerous models, YAPer-ORF prominently drove cell proliferation in a CCND1-dependent manner. Notably, cardiac-specific genetic knock-in of the human YAPer-ORF in mice significantly increased heart size through increased cardiomyocyte proliferation, underscoring the role of YAPer-ORF in cell proliferation. Moreover, treatment with an anti-YAPer-ORF neutralizing antibody effectively suppressed uveal melanoma growth, highlighting the therapeutic potential of targeting YAPer-ORF. These findings collectively establish YAPer-ORF as a critical regulator of YAP activity, further highlighting the disruption of YAPer-ORF activity as a potential therapeutic strategy against YAP-driven human cancers and developmental diseases.

## Introduction

The Hippo-YAP pathway is an evolutionarily conserved signaling cascade that performs pivotal functions in diverse physiological and pathological processes [1]. As a transcriptional coactivator, YAP is central to the Hippo-YAP signaling pathway. Importantly, the dephosphorylation of YAP at serine 127 and 397 facilitates its translocation to the nucleus, where YAP interacts with TAZ/TEAD to activate the transcription of downstream target genes, thereby initiating developmental disorders and cancers [2, 3]. Consequently, strategies aimed at inhibiting YAP activity have emerged as potential therapeutic interventions, either by perturbing the YAP-TEAD interface or by targeting the core kinases in the pathway. However, existing approaches to inhibit YAP, such as verteporfin, have exhibited both systemic proteotoxic effects and cytotoxicity, thereby limiting their clinical viability [4]. Thus, there is an unmet need to develop therapeutic methods that are specifically tailored to counteract hyperactive YAP while preserving its normal physiological activity.

Noncoding RNAs, particularly long noncoding RNAs (lncRNAs), prominently contribute to the regulation of Hippo-YAP signaling and its associated disorders [5, 6]. LncRNAs are autonomously transcribed RNA molecules over 200 nucleotides in length that do not encode protein products [7, 8]. Recent investigations have revealed the presence of small open reading frames (smORFs) within a subset of lncRNAs that have the potential to encode small peptides or proteins [9, 10]. Notably, smORF primary sequences exhibit greater conservation and higher expression levels than do introns and other lncRNA regions, suggesting that lncRNAs might perform more substantial biological functions than do previously recognized lncRNAs [11]. For example, lncRNA-encoded smORFs such as myoregulin and DWORF serve as regulators of muscle performance by inhibiting calcium pump activity [12, 13]. Additionally, the NoBody peptide, which is translated from the lncRNA LINC01420, interacts with mRNA decapping proteins to govern the abundance of P-bodies [14]. Moreover, the SPAR peptide derived from LINC00961 impedes mTORC1 activation and muscle regeneration [15]. Previous findings indicate that the VGLL4 peptide or YAP (D94A) mimics exert antagonistic effects on YAP by competing with YAP for binding to TEADs [16, 17]. Our understanding of the functions and mechanisms underlying the ability of lncRNA-derived smORFs to directly modulate YAP activity in disease remains limited.

Uveal melanoma (UM) is the most prevalent primary ocular malignancy in adults and is characterized by a high rate (83%) of GNAQ/11 mutations that drive the aberrant activation of YAP signaling [18–20]. Previous studies have shown that GNAQ/11 mutations activate YAP via the Trio-Rho/Rac signaling pathway, promoting actin polymerization in the cytoplasm [21]. Here, we elucidated the mechanism through which YAPer-ORF competes with PRP4K, a well-established kinase responsible for phosphorylating YAP at multiple sites within the nucleus [22]. In this study, we integrated transcriptomic data from neurodevelopmental disorders and diverse sets of data from various cancers and identified *LINC01315* as a unique shared gene. LINC01315, which encodes a small protein named YAPer-ORF, has emerged as a critical factor that promotes cell growth in cardiovascular development and cancer models, including UM. Mechanically, YAPer-ORF was found to preferentially interact with GNAQ/11 mutants, leading to the aberrant activation of YAP signaling. YAPer-ORF resulted in reduced YAP phosphorylation levels but enhanced nuclear retention. Notably, YAPer-ORF facilitated the nuclear translocation of mutated GNAQ/11 (Q209L), enabling further suppression of PRP4K at the transcription level. Importantly, the genetic depletion or neutralization of YAPer-ORF abolished its proliferative effects, contingent upon YAP signaling. Our findings provide novel insights into the regulatory mechanisms of YAP activity mediated by lncRNA-encoded small proteins, underscoring the therapeutic potential of YAPer-ORF-neutralizing antibodies in the treatment of developmental disorders and cancers characterized by dysregulated YAP activity.

## Materials and methods

### Data capture and meta-analysis

Disruptions in the Hippo signaling pathway are implicated in various cancers, including colorectal carcinoma, triple-negative breast carcinoma, and esophageal squamous cell carcinoma [23–25]. We extracted transcriptomic data from the Gene Expression Omnibus (GEO) database for patients with colorectal carcinoma (GSE39582, GSE41328), triple-negative breast carcinoma (GSE7904, GSE3744, GSE65194, GSE76275, GSE45827, GSE21653), and esophageal squamous cell carcinoma (GSE161533, GSE45670). Differential gene expression was identified in each cancer type using the R package ‘DESeq2’, applying a trimming criterion of false discovery rate (FDR) < 0.05 and |log2 (fold change)|>2. We then used Venn diagram analysis to identify the unique gene LINC01315. D’haene et al. suggested that LINC01315 may be involved in neurodevelopmental and central nervous system disorder pathogenesis through the integration of multi-omics data, including epigenetics, transcriptomics, and whole-genome sequencing [26]. Building on these findings, we hypothesize that LINC01315 may have a conserved role in embryonic development and tumorigenesis, and we aim to investigate whether this gene modulates YAP signaling activity.

### Plasmid DNA constructs

To knock down LINC01315 and other genes, DNA sequences for shRNAs were designed by the GPP Web Portal (https://portals.broadinstitute.org/gpp/public/). DNA oligos of shRNA sequences for targeted RNAs (shLIN01315#1: CGCTCTGAAGGATCTCGAGAG; shLIN01315#2: GACCCTCGAGCTGCAGCTCT) or scramble shRNAs were individually cloned and inserted into the pLKO.1-TRC vector [27]. eGFP fusion protein constructs (ORF-GFPmut) and HA fusion protein constructs (ORF-4×HA) with the LINC01315 ORF were generated as previously described [28]. To construct plasmids to overexpress LINC01315 or ORF, the full-length LINC01315 CDS or ORF sequence was amplified from the HEK293T cDNA library and cloned and inserted into the pcDNA3 vector. All plasmids were confirmed by DNA sequencing. The primers used for plasmid construction are listed in Table 1.

**Table 1:** The primer sequences used in this study.

Name	Sequence (5’ > 3’)
hGAPDH-RT-F	TGCACCACCAACTGCTTAGC
hGAPDH-RT-R	GGCATGGACTGTGGTCATGAG
18sRNA-RT-F	AACCCGTTGAACCCCATT
18sRNA-RT-R	CCATCCAATCGGTAGTAGCG
Linc#1-RT-F	CTGTTGAGCCTGTGGAGAC
Linc#1-RT-R	GCTGTTGAAGCAGTGATTTGAG
Linc#2-RT-F	GAATCCGGGGGTGGTCAAGACG
Linc#2-RT-R	GGTAGGGGGAAAACGCTGCC
Linc#3-RT-F	GTGGCCGCAGGAGGATTTAC
Linc#3-RT-R	GAAAACGCTGGCCCGCAC
Total-LINC-RT-F	GACATATCAGATAGATTCTGAAATC
Total-LINC-RT-R	TAAAATGATGTCCAAAAGGTTTG
PRP4K-RT-F	AAGAGCCAAGAGCCGATCCT
PRP4K-RT-R	CGGGTTGGAGACCATCTATTGA
GNA11-RT-F	TCATCGAGTACCCTTTCGACC
GNA11-RT-R	CATGATGGATGTCACGTTCTCA
CDC42-RT-F	GATTACGACCGCTGAGTTATCC
CDC42-RT-R	GTTATCTCAGGCACCCACTTT
LATS1-RT-F	TTACCAAGATCCTCGACGAGAG
LATS1-RT-R	CACATTCCCTGGTTTCATGCT
LATS2-RT-F	GAAGTGAACCGGCAAATGCTG
LATS2-RT-R	GAAGTGAACCGGCAAATGCTG
CDC14A-RT-F	ACGCCCCTGAAGCCTACTT
CDC14A-RT-R	AGAAGAGGTCATAGTGCTCGAAG
CK1-RT-F	AGATGTTGTCACGACTCCAAAAA
CK1-RT-R	CTCCTGGATCGGCAGTCAT
CDK1-RT-F	GAGAAGGTACCTATGGAGTTGTG
CDK1-RT-R	CCCTTCCTCTTCACTTTCTAGTC
CCND1-RT-F	GTTCGTGGCCTCTAAGATGAAG
CCND1-RT-R	GATGGAGTTGTCGGTGTAGATG
YAP1-RT-F	CAGACAGTGGACTAAGCATGAG
YAP1-RT-R	CAGGGTGCTTTGGTTGATAGTA
LINC01315-R	CGGAATTCCAAAGCTTCAGGTCTGATTTATTAG
LINC01315-F	GCTCTAGACTGAGGGCCAGAGAAAGGGAG
ORF-F	CGTTTAAACGGGCCCTCTAGAATGGTGCAGGCGGGACCC
ORF-R	CTCGTCGCTCTCAATTCTAGATCGCTCAGCAGCCAGCCA

### Cell culture and treatments

HEK293T (#CVCL_1926), HeLa (#CVCL_0030), and U-251 MG (#CVCL_0021) cells were obtained from CCTCC and were cultured in DMEM supplemented with 10% FBS and 1% penicillin/streptomycin (Thermo Fisher Scientific). The UM cell lines 92.1 (#CVCL_8607), MEL202 (#CVCL_C301), MEL290 (#CVCL_C304), MEL270 (CVCL_C302), OMM2.3 (CVCL_C306) and the adult retinal pigment epithelial cell line ARPE19 (#CVCL_0145) were obtained from ECACC and were cultured in RPMI 1640 supplemented with 10% FBS and 1% penicillin/streptomycin (Thermo Fisher Scientific). The cells were incubated at 37 °C in 5% CO2. All the cell lines were authenticated by STR profiling and tested for mycoplasma contamination, and the results were negative.

To establish stable expressing cell lines, the cells were infected with 5 µL of concentrated corresponding lentivirus or 5 mg/mL polybrene (Sigma‒Aldrich) for 24 h. The cells were infected a second time with prewarmed medium containing the contents described above on the second day, and then, cell selection was performed with puromycin for one week. Protein or total RNA was collected for subsequent analyses.

### Cell transfection and lentivirus production

The full-length LINC01315 sequence and the ORF-4×HA sequence were separately cloned and inserted into pCDH-CMV-MCS-EF1-Puro from Dr. Liang Yan (Fudan University). To produce lentiviral particles, HEK293T cells were cotransfected with the vector described above or pLKO.1 shRNA targeting LINC01315 and the lentiviral vector packaging system using the Lipofectamine 2000 Reagent (Invitrogen) according to the manufacturer’s instructions. The supernatant containing viral particles was harvested twice at 48 h or 72 h after transfection and then filtered through a Millex-GP filter unit (0.22 mm pore size, Millipore). Viral particles were concentrated approximately 100-fold with Lenti-Concentin Virus Precipitation Solution (ExCell Bio), resuspended in PBS containing 0.1% BSA, and stored at −80 °C until use.

### Mouse experiments

All experimental procedures were approved by the Institutional Animal Care and Use Committee of Shanghai Jiao Tong University (A2020008). The sample size required for the animal experiments was calculated, and the animals were randomly assigned to different groups using the power analysis method. There was no blinding of researchers or participants. BALB/c nu/nu female mice (6–8 weeks) were used for all in vivo xenograft studies. In vivo tumor growth assays were performed as previously described [29]. Briefly, 92.1 cells were subcutaneously injected into the dorsal flank of each BALB/c nu mouse (*n* = 6). After three weeks, the mice were euthanized, and the tumors were dissected and weighed. Tumor-bearing mice were treated with vehicle or an anti-YAPer-ORF antibody every 3 days. Tumor size was measured every 3 days, and tumor volume was determined using the following formula: (width × width × length)/2.

### Reagents and antibodies

Peptide synthesis (*CSRRIPKAEVGSPGD*) and anti-YAPer-ORF monoclonal antibody preparation were performed by GenScript (Nanjing, China). The following primary antibodies were used: anti-GAPDH (#30201ES20, Yeasen, 1:3000 dilution), anti-HA (#30701ES20, Yeasen, 1:3000 dilution), anti-FLAG (#14793, Cell Signaling Technology, 1:1000 dilution), anti-LaminA/C (#4777, Cell Signaling Technology, 1:2000 dilution), anti-p-YAP1 ser127 (#13008, Cell Signaling Technology, 1:2000 dilution), anti-p-YAP1 ser397 (#13619, Cell Signaling Technology, 1:2000 dilution), anti-PRP4K (#8577, Cell Signaling Technology, 1:2000 dilution), anti-GNAQ (#sc-136181, Santa Cruz, 1:2000 dilution), anti-GNA11 (#sc-390382, Santa Cruz, 1:2000 dilution), anti-YAP1 (#sc-101199, Santa Cruz, 1:2000 dilution), anti-LAST1 (#3477, Cell Signaling Technology, 1:2000 dilution), anti-MST1 (#14946, Cell Signaling Technology, 1:2000 dilution), anti-MST2 (#2017, Cell Signaling Technology, 1:2000 dilution), anti-MOB1 (#13730, Cell Signaling Technology, 1:2000 dilution), anti-p-MOB1 Thr35 (#8699, Cell Signaling Technology, 1:2000 dilution), anti-TEAD1 (#12292, Cell Signaling Technology, 1:2000 dilution), anti-CCND1 (#26939-1-AP, Proteintech, 1:1000 dilution), anti-CCND3 (#26755-1-AP, Proteintech, 1:1000 dilution), anti-CDK4 (#11026-1-AP, Proteintech, 1:1000 dilution), anti-CDK2 (#10122-1-AP, Proteintech, 1:1000 dilution), anti-p18 INK4C (#ab192239, Abcam, 1:2000 dilution), anti-p21 (#10355-1-AP, Proteintech, 1:1000 dilution), and anti-p27 KIP (#ab32034, Abcam, 1:2000 dilution).

#### Reverse transcription and real-time PCR

Total RNA was extracted with TRIzol reagent (Vazyme) and then reverse transcribed into cDNA using a HiScript III First Strand cDNA Synthesis Kit (Vazyme). The resulting cDNA was then subjected to RT–PCR analysis with ChamQ SYBR qPCR Master Mix (Vazyme) in a StepOnePlus real-time PCR system (Applied Biosystems). The primers used in this study are listed in Table 1.

### RNA-seq data processing

RNA was harvested using TRIzol reagent (Vazyme), and 5 µg of total RNA was used for the construction of the sequencing libraries. RNA libraries for RNA sequencing were prepared using the VAHTS Universal V10 RNA-seq Library Prep Kit for Illumina following the manufacturer’s instructions. The sequence reads were trimmed for adaptor sequences/low-quality sequences using CLC Genomics Workbench (version 11.0.1, parameter-quality limit: 0.05). The trimmed sequence reads were mapped to GRCh38.p13/hg38 using CLC Genomics Workbench (parameters - mismatch cost: 2; insertion cost: 3; deletion cost: 3; length fraction: 0.8; and similarity fraction: 0.8). Read count extraction and normalization were performed using the CLC Genomics Workbench. Gene expression levels were calculated with fragments per kilobase of transcript per million mapped reads (FPKM) values by normalizing gene counts from the CLC Genomics Workbench.

### Circular dichroism spectroscopy and imaging experiments

RNA samples were prepared at a strand concentration of 4 µM in RNase-free water and degassed buffers containing 10 mM Tris-HCl, pH 7.4, and 100 mM KCl. All the samples were annealed by heating at 90 °C for 10 min and then slowly cooled to 5 °C at a controlled rate of 0.2 °C min^–1^. Circular dichroism spectroscopy was performed using a Jasco J-810 spectropolarimeter equipped with a Peltier temperature controller. The sample was placed in a quartz cuvette with an optical path length of 1 mm, transferred to the spectropolarimeter and allowed to equilibrate at 20 °C for 10 min. Five CD scans, over the wavelength range of 220–320 nm, were performed at 50 nm min^–1^ with a 2-s response time, 1-nm pitch and 1-nm bandwidth, and the average was taken. The data were zero-corrected at 320 nm. For the imaging experiments, 4 µM RNA molecules were dissolved in 200 µl of structure buffer (10 mM Tris-HCl, pH 7.4, and 100 mM KCl), and 10 µM DASPMI fluorescent agent was added to the sample after annealing. All the samples were exposed to UV light (λ = 312 nm) for a few seconds, and then, images were acquired.

### Luciferase reporter assays

The LINC01315 ORF sequence was cloned and inserted into the pBIND vector, and the corresponding cDNA sequence of wild-type or mutated GNAQ/11 was subsequently cloned and inserted into the pACT vector. The interaction between ORF and GNAQ/11 was assessed using the CheckMate™ Mammalian Two-Hybrid System (Promega) according to the manufacturer’s instructions. The luciferase activities of the reporters were measured using the Dual Luciferase Reporter Assay System (Promega).

### Immunofluorescence staining

UM specimens from patients were provided by the Clinical Specimen Resource Center, Shanghai Ninth People’s Hospital, Shanghai JiaoTong University School of Medicine. Tissue sections or HeLa cells cultured on poly-L-lysine-coated coverslips were transfected with a series of ORF‒GFP fusion plasmids for 24 h, and GFP fluorescence was directly visualized via a Nikon Eclipse Ti‒U fluorescence microscope. Cells transfected with ORF-4×HA fusion plasmids were fixed with 4% formaldehyde in phosphate-buffered saline (PBS) for 20 min and permeabilized with 0.05% Triton X-100 for 10 min. The cells were blocked with 3% BSA-containing PBS for 30 min and incubated with the indicated antibodies in 3% BSA-PBS for 1 h at room temperature. The reaction was visualized with Alexa-labeled secondary antibodies (Invitrogen). The samples were mounted in PBS buffer containing Hoechst 33342 (Invitrogen) for nuclear staining. Images were acquired with a ZEISS LSM 880 controlled by ZEN software.

### Subcellular fractionation

NE-PER Nuclear and Cytoplasmic Extraction Reagents (Thermo Fisher) were used according to the manufacturer’s instructions. Briefly, after 24 h, HeLa cells transfected with ORF-4×HA fusion plasmids were washed in ice-cold PBS, suspended in CER I and II buffer and homogenized with a Dounce homogenizer. The supernatant, as the cytosolic fraction, was centrifuged at 12,000 rpm for 10 min at 4 °C, and the pellet (nuclear fraction) was subsequently suspended in ice-cold NER. The resulting extracts were analyzed by immunoblotting.

### Western blotting

Protein samples were subjected to SurePAGE on 4‒20% Bis‒Tris acrylamide gels (GenScript) in Tris‒MOPS‒SDS running buffer. PVDF membranes were incubated first with primary antibodies and subsequently with secondary HRP-tagged antibodies (Cell Signaling), and the signals were visualized with enhanced chemiluminescence (ECL) (Thermo Fisher). Bands were quantified by densitometric analysis using the ImageJ software program.

### Immunoprecipitation and mass spectrometry (MS)

The ORF-4×HA fusion protein was expressed in HeLa cells, and whole-cell lysates were prepared in ice-cold lysis buffer (40 mM HEPES (pH 7.4), 10 mM β-glycerol phosphate, 10 mM pyrophosphate, 0.3% CHAPS, 2.5 mM MgCl2 and EDTA-free protease inhibitor). The soluble fractions from the cell lysates were immunoprecipitated with anti-HA agarose-conjugated beads (Abmart) for 3 h at 4 °C and then washed three times with lysis buffer. Immunoprecipitated proteins were eluted with elution buffer (0.2 M glycine, pH 2.2) and subjected to mass spectrometry analysis via ultrafast Xtreme MALDI-TOF/TOF (Bruker) or SDS‒PAGE. MS was performed by professionals at the State Key Laboratory of Genetic Engineering, Fudan University.

### Colony formation

UM cell lines infected with lentivirus were seeded in 6-well culture plates, cultured in RPMI 1640 medium supplemented with 10% FBS for two weeks, fixed with methanol and stained with crystal violet solution. The numbers of colonies containing ≥30 cells were counted under a microscope (*n* = 3).

### Cell growth assay

A total of 4000 cells were seeded in 96-well culture plates and cultured in RPMI 1640 medium supplemented with 10% FBS. The number of cells was counted at 12, 24, 36, 48, and 60 h postseeding.

### Statistical analysis

All experiments were repeated 3 times unless stated in the figure legend. Statistical significance for comparisons was generally assessed by Student’s *t* test, with the exceptions described below. The data are presented as means ± SEMs except where stated elsewhere. Differences for which *p < 0.05 or **p < 0.01 were considered statistically significant.

## Results

### Identification of LINC01315, which deregulates YAP activity through an encoded small protein

**Fig. 1:**
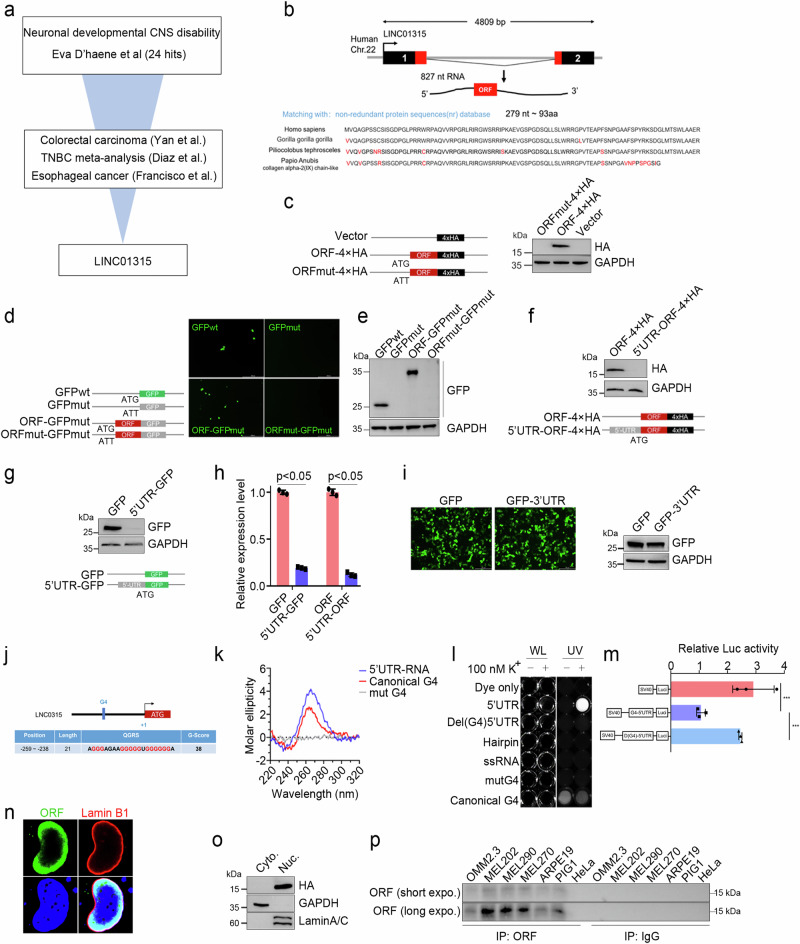
Identification of LINC01315, which encodes a small protein that deregulates YAP activity. **a** Schematic illustration of the process for screening differentially expressed lncRNAs (fold change >2, *P*  <  0.05) using 11 independent datasets (GSE39582, GSE41328, GSE7904, GSE3744, GSE65194, GSE76275, GSE45827, GSE21653, GSE161533, GSE45670 and Eva D’haene). **b** Genomic structural features and alignment of the amino acid sequence of LINC01315-ORF. The upper section indicates ORF genetic locations and transcriptional sequence features, and the bottom section represents ORF amino acid sequence conservation in four species of primates. **c**–**e** Diagram of the HA and GFP fusion constructs and the fusion protein expression levels. In the mutation fusion constructs, the start codon ATG of the LINC01315-ORF is changed to ATT (**e**, left and **d**, left). All the fusion constructs were transfected into HeLa cells for 24 h, and the fusion protein levels were determined via western blotting with anti-GFP (**d**, right) and anti-HA (**c**, right) antibodies and GFP fluorescence (**d**, middle). Scale bar: 500 μm. Internal reference: GAPDH. The LINC01315 5’ UTR significantly inhibits ORF translation. Diagram of the 5’UTR-fusion constructs (**f**, bottom and **g**, bottom). The fusion protein level was assessed by western blotting with anti-GFP (**f**, upper) and anti-HA (**g**, upper) antibodies. **h** 5’ UTR-GFP mRNA levels were determined by RT‒qPCR. **i** The LINC01315 3’ UTR did not affect ORF translation. GFP fluorescence (**i**, upper) and western blotting were used to assess protein expression levels. **j-m** Identification and functional verification of 5’ UTR G-quadruplexes. Schematic structure of the 5’ UTR G-quadruplex (**j**). Circular dichroism (**k**) and imaging (**i**) experiments to determine the structure. CD spectrum of the 5’ UTR at a strand concentration of 4 µM in 10 mM Tris-HCl, pH 7.4, and 100 mM KCl at 20 °C. UV profiles of the 5’ UTR at a 4 µM strand concentration in 10 mM Tris-HCl, pH 7.4, 100 mM KCl and 10 µM DASPMI fluorescent agent. WL: white light. UV: ultraviolet (λ = 312 nm). 5’ UTR G-quadruplexes repressed fluorescent reporter gene activity (**m**). **n** Immunofluorescence staining of the nuclear lamina markers LAMIN A/C (red) and ORF (green) in HeLa cells. **o** ORF is expressed in the nucleus. GAPDH and LaminA/C are markers of the cytoplasm and nucleus. **p** Analysis of endogenous ORF expression. Endogenously expressed ORF was enriched with an anti-ORF antibody, and the expression level was assessed via western blotting. Control: mouse IgG. The error bars represent the SDs of triplicate experiments. All *p* values were calculated using two-tailed unpaired Student’s *t* test; *p < 0.05, ***p < 0.001.

Hippo-YAP signaling plays a prominent role in various tumors, as well as in the development of diseases [1, 2]. To identify whether lncRNAs function in dysregulated Hippo signaling in tumors and diseases, we utilized transcriptomic data from various cancers to identify differentially expressed lncRNA genes and then integrated multiomics data from patients with developmental disorders to identify potentially pathogenic lncRNA genes and narrowed them down to the sole gene LINC01315. An open reading frame (ORF) spanning 279 nucleotides in the trans-exon region, encoding 93 amino acids was predicted by the CPC 2.0 database (https://cpc2.gao-lab.org/). Importantly, we confirmed that this ORF is evolutionarily conserved in vertebrates, with the protein sequence exhibiting high conservation among nonhuman primates (Fig. 1b).

Western blot analysis confirmed the stable expression of the ORF in HEK-293T cells transfected with the ORF-HA or ORF-GFP fusion protein (Fig. 1c–e). However, when HEK-293T cells were transfected with a tagged ORF containing the upstream 274 base pairs in the 5’ untranslated region (UTR), ORF expression was not detected. Similarly, GFP expression disappeared after we cloned the 5’ UTR of LINC01315 at the 5’ end of the GFP coding sequence (Fig. 1f, g). The RT‒PCR results revealed a significant reduction in the RNA levels of the ORF and GFP genes in the presence of the 5’ UTR of LINC01315, indicating its inhibitory effect on RNA transcription (Fig. 1h). In the control, the expression of GFP was unaffected by alterations in the 3’ UTR sequence (Fig. 1i). To explore how the 5’ UTR regulates ORF transcription, we first predicted, using online tools, secondary structures of the 5’ UTR sequence; the data revealed that the position located in the interval 259–238 nt upstream of the start codon is capable of G-quadruplex formation (Fig. 1j). Then, we performed circular dichroism and imaging, which confirmed the presence of G-quadruplexes (Fig. 1k‒l). Finally, the results of the fluorescence reporter gene activity assay indicated that G-quadruplexes are responsible for suppressing ORF expression (Fig. 1m).

Next, the computational methods PSORT and NLStradamus were employed to identify potential subcellular localizations of YAPer-ORF. The results indicated that YAPer-ORF localized to the nucleus, with a 71.6% probability and 94.1% reliability [30, 31]. These predictions highlighted the presence of two putative conserved motifs resembling nuclear localization signals (NLSs). Furthermore, immunofluorescence staining and nucleoplasmic protein profiling of HeLa cells expressing YAPer-ORF with an HA tag revealed punctate staining predominantly located within the nucleus (Fig. 1n, o); the immunofluorescence and western blot results obtained via the use of customized in-house antibodies instead of exogenous anti-HA antibodies were consistent with those results (Supplementary Fig. 1a, b). To select an appropriate tumor cell line for the detection of endogenous ORF expression, we initially analyzed the expression of LINC01315 in cancers with YAP signaling dysregulation and its correlation with patient prognosis. The results demonstrated a robust association between LINC01315 expression and the prognosis of uveal melanoma (UM) patients, suggesting that LINC01315 may play a more significant role in this cancer type (data not show). Consequently, we prioritized the human-derived UM cell lines OMM2.3, Mel202, Mel290, and Mel270 for the detection of endogenous ORF expression and the normal retinal epithelial cell lines ARPE19, PIG1, the carcinoid cancer cell line HeLa as control. The results revealed that YAPer-ORF was specifically expressed in UM cells (Fig. 1p).

### YAPer-ORF promotes aberrant cell proliferation and growth

**Fig. 2:**
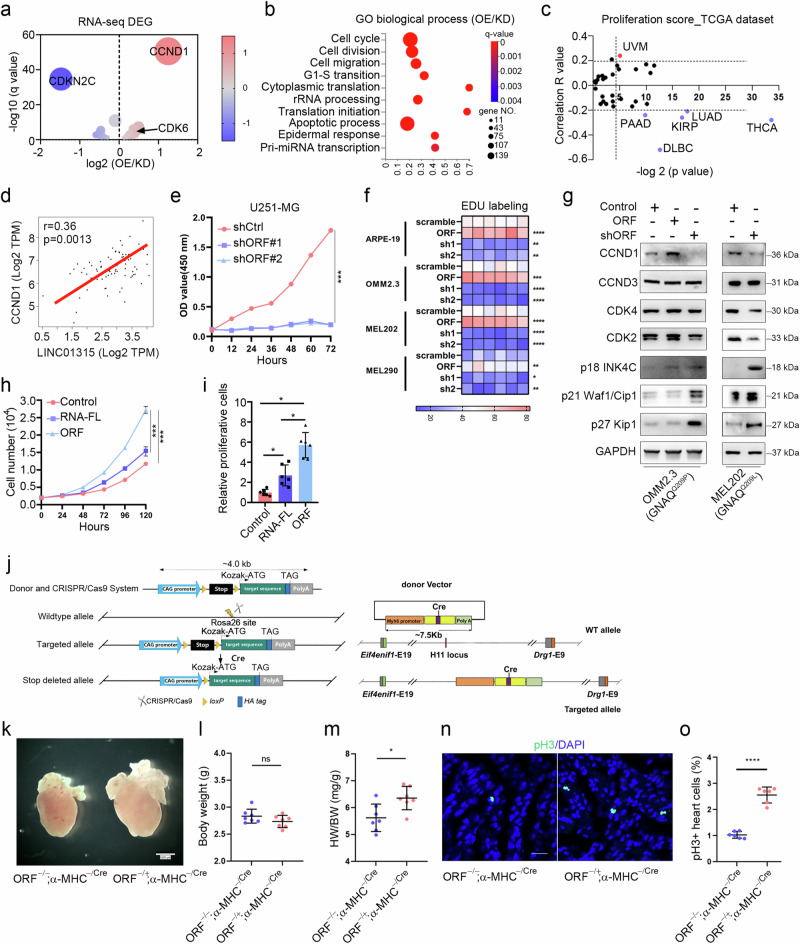
YAPer-ORF promotes aberrant cell proliferation and growth. Volcano plot of the cell cycle terms (**a**) and Gene Ontology analysis (**b**) of differentially expressed genes in OMM2.3 cells with overexpression/knockdown of YAPer-ORF. “GO biological process” category denotes the biological processes identified through Gene Ontology (GO) analysis. “OE” refers to the cell subset with overexpression of YAPer-ORF but not LINC01315, “KD” to the subset with knockdown of YAPer-ORF and LINC01315, and “OE/KD” signifies the comparative analysis between the overexpression and knockdown subsets. **c** Proliferation score for LINC01315 in different cancer types. The proliferations score was determined by calculating the mRNA expression of MIK67 in the online database GEPIA 2.0 (http://gepia2.cancer-pku.cn/) and to evaluate the Spearman’s coefficient of LINC01315 and MIK67 (correlation R value). **d** Correlation between LINC01315 and CCND1. **e** Knockdown of ORF expression significantly inhibited glioma cell growth. **f** Heatmap of the proliferation ability (EdU labeling) of normal and UM cells with overexpression/knockdown of YAPer-ORF. Each box represents the biological replicate of the experiment under each condition (a total of 6 replicates). **g** Western blot analysis of the protein levels of CCND1, CCND3, CDK4, CDK2, p18 INK4C, p21 Waf1/Cip1 and p27 Kip1 in OMM2.3 cells with overexpression/knockdown of YAPer-ORF or scramble control. **h**, **i** ORF but not RNA promoted cell growth. RNA-FL: full-length RNA. The error bars represent the SDs of triplicate experiments. *p < 0.05, **p < 0.01, ***p < 0.001. **j** Schematic illustration of the strategy for α-MHC-derived knockout in a mouse model. **k**‒**m** Heart weight/body weight ratio (**m**) and body weight (**l**) of P0.5 neonatal heterozygous YAPer-ORF-knock-in (KI) and control mice. Immunofluorescence staining (**n**) of pH3 in heart sections from P0.5 neonatal mice and quantification (**o**) of the pH3-positive cells in the heart.

To investigate how YAPer-ORF effects on cellular function, we performed RNA sequencing analysis of YAPer-ORF overexpressed and knockdown OMM2.3 cells. Gene ontology and KEGG analysis of the RNA sequencing results revealed that the most significantly enriched pathways involved cell cycle processes, including cell division, cell migration, and the G1 to S transition of the mitotic cell cycle (Fig. 2a, b). In the cell cycle pathway, *CCND1* was the most significantly upregulated gene, whereas *CDKN2C* (*p18INK4C*) was the most significantly downregulated gene (Fig. 2a). To investigate the impact of YAPer-ORF on the cell cycle, we analyzed the proliferation score (*MIK67*) in different cancer types via the GEPIA2.0 database. Notably, UM exhibited the highest score among all cancer types, suggesting a crucial role of YAPer-ORF in regulating UM proliferation (Fig. 2c). Additionally, we observed a positive correlation between the expression levels of LINC01315 and *CCND1* mRNA in the TCGA-UM dataset (Fig. 2d).

LINC01315 knockdown significantly inhibited the growth of glioma cells (Fig. 2e). EdU labeling assays demonstrated that YAPer-ORF overexpression led to a significant increase in cell proliferation in multiple UM cell lines (MEL202, MEL270, MEL290, OMM2.3, and 92.1 cells) as well as in normal cells (ARPE19 and PIG1 cells) (Fig. 2f). Conversely, YAPer-ORF knockdown attenuated the proliferation of both UM and normal cells (Fig. 2f and Supplementary Fig. 2a). Furthermore, TUNEL assays revealed that the level of apoptosis remained unchanged in both control and YAPer-ORF-knockdown cells (Supplementary Fig. 2a, b), suggesting that the aforementioned effects were largely independent of cell apoptosis. Western blot analysis revealed that YAPer-ORF knockdown substantially reduced the protein expression of CCND1 and increased the expression of p18 INK4C in OMM2.3 and MEL202 cells (Fig. 2g). Conversely, YAPer-ORF overexpression increased CCND1 expression but decreased p18 INK4C expression (Fig. 2g). These findings indicate that the impact of YAPer-ORF on cell proliferation strongly hinges on CCND1. To discern whether these effects were solely attributed to the YAPer-ORF protein or LINC01315 itself, we overexpressed YAPer-ORF or the corresponding full-length lncRNA in glioma cells. The results revealed that, compared with its full-length counterpart, YAPer-ORF had a significantly greater effect on cell growth, indicating that the cell growth-promoting effects of LINC01315 primarily stemmed from YAPer-ORF (Fig. 2h, i).

Given that YAP functions as a key regulator of organ growth, YAP deletion in the embryonic heart hampers cardiomyocyte proliferation and leads to myocardial hypoplasia [32]. To investigate the potential of YAPer-ORF in enhancing cardiomyocyte proliferation in vivo, we assessed the impact of cardiac-YAPer-ORF expression in a genetic mouse model. Given that mice lack a YAPer-ORF homolog, we generated a strain of Rosa26-YAPer-ORF knock-in mice that harbored a Lox-STOP-Lox (LSL)-YAPer-ORF cassette under the control of the CAG promoter (Rosa26-CAG-LSL-YAPer-ORF). These mice were then crossed with H11-knockout α-myosin heavy chain (H11-α-MHC)-Cre mice (H11-α-MHC^Cre/Cre^), resulting in YAPer-ORF^−/+^;α-MHC^Cre/Cre^ mice (Fig. 2j). The resulting embryos developed normally and were born in Mendelian ratios. Verification of YAPer-ORF expression was performed in cultured neonatal mouse cardiomyocytes (Fig. 2j). The YAPer-ORF^−/+^;α-MHC^Cre/Cre^ mice presented enlarged hearts and an elevated heart weight/body weight ratio, which could not be attributed to cardiomyocyte hypertrophy, as there was no significant difference in cardiomyocyte size between the YAPer-ORF^−/+^;α-MHC^Cre/Cre^ and control groups (Fig. 2k, m and Supplementary Fig. 3a, b). Immunostaining for phosphorylated serine 10 on histone H3 (pH3), a marker of the mitotic phase of the cell cycle, revealed a substantial increase in cardiomyocyte proliferation (Fig. 2n, o). Collectively, these findings indicate that YAPer-ORF can drive the proliferation of cardiomyocytes.

### YAPer-ORF interacts with GNAQ/11 mutants to activate YAP signaling

**Fig. 3:**
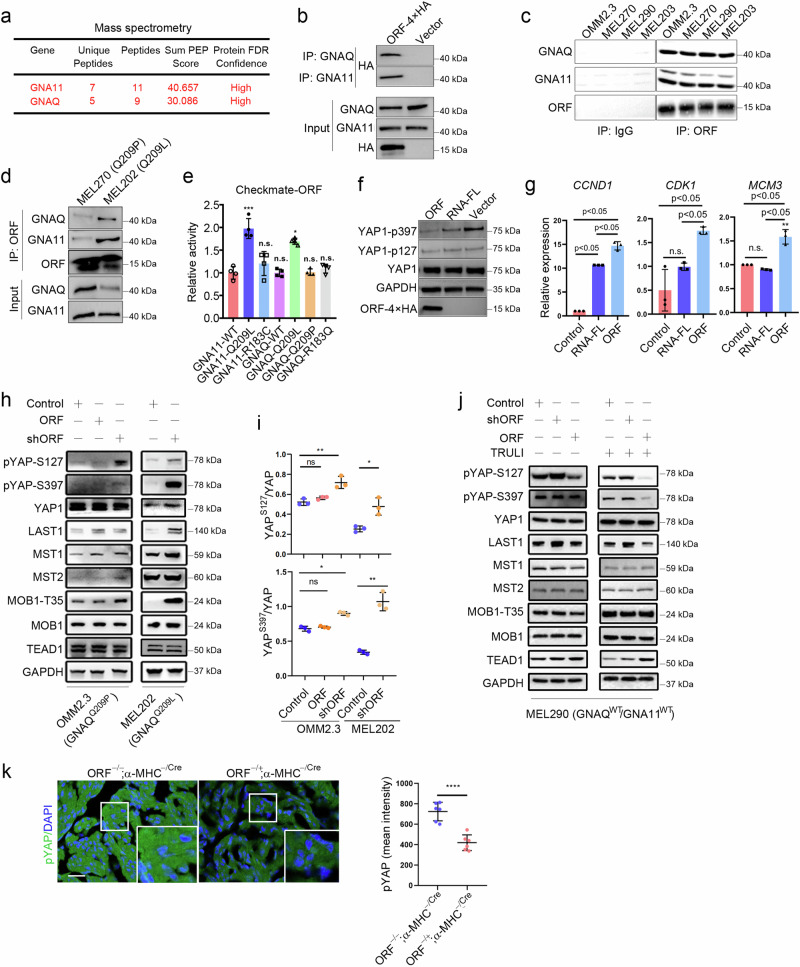
YAPer-ORF interacts with GNAQ/11 to activate YAP signaling. **a**–**d** YAPer-ORF interacts specifically with GNAQ/11. Mass spectrometry identification of ORF-interacting proteins (**a**). The ORF-4×HA fusion construct was transfected into HEK-293T cells for 24 h, the fusion protein was enriched with an anti-HA antibody, and the GNAQ/11 expression level was assessed via western blotting with anti-GNAQ or anti-GNA11 antibodies (**b**). Endogenously expressed YAPer-ORF was enriched with anti-ORF, and the GNAQ/11 expression level was assessed via western blotting (**c**, **d**). **e** The interaction between YAPer-ORF and the GNAQ/11-Q209L mutant was markedly elevated, as determined by the Checkmate assay. GNA11-WT and GNAQ-WT are wild-type proteins, and GNA11-Q209L, GNA11-R183C, GNAQ-Q209L, GNAQ-Q209P, and GNAQ-R183Q are mutated proteins. **f** YAPer-ORF overexpression significantly decreased YAP1 phosphorylation levels and activated YAP signaling activity. YAP signaling activity was determined via western blotting with anti-YAP1-p397 and anti-YAP1-p127 antibodies. RNA-FL: full-length RNA. Internal reference: GAPDH and YAP1. **g** YAPer-ORF overexpression upregulated the mRNA expression of YAP signaling target genes. The data in **e** and **g** are presented as means ± SDs. The error bars represent the SDs of triplicate experiments. The *p* values were calculated using two-tailed unpaired Student’s *t* test; *p < 0.05, **p < 0.01, ***p < 0.001. Western blot analysis (**h**, **j**) and quantification (**i**) of protein levels of YAP^S127^, YAP^S397^, YAP, LAST1, MST1, MST2, MOB1^T35^, MOB1 and TEAD in OMM2.3, MEL202, and MEL290 cells with overexpression/knockdown of YAPer-ORF or scramble control and treated with/without TRULI (**j**). **k** Immunofluorescence staining (left) of pYAP in heart sections from P0.5 neonatal mice and quantification (right) of the intensity of pYAP in the heart.

To explore the molecular mechanism by which YAPer-ORF regulates YAP signaling activity to promote cell proliferation, we first utilized immunoprecipitation and mass spectrometry to determine potential YAP activity regulators corresponding to YAPer-ORF. As a result, we identified GNAQ/11 via mass spectrometry and confirmed its specific binding via semiendogenous and endogenous methods. GNAQ/11 is a highly mutated protein in UM patients, and 95% of patients harbor a site-specific variant of Q209. Therefore, we investigated whether the GNAQ/11 Q209 mutation affects interactions with YAPer-ORF via a checkmate assay, and the results revealed a notable increase in the interaction of YAPer-ORF with the GNAQ/11 Q209L mutation (Fig. 3a–e). The finding that YAP signaling activity is affected by YAP phosphorylation and the expression of downstream target genes revealed that, rather than full-length RNA, YAPer-ORF plays a prominent role as a regulator of YAP signaling (Fig. 3f, g). Specifically, we assessed YAPer-ORF-induced YAP activation in three cell lines (OMM2.3, MEL202, and MEL290 cells) derived from primary or metastatic UM samples harboring GNAQ-Q209P, GNAQ-Q209L, or wild-type GNAQ/11, respectively. YAPer-ORF knockdown in OMM2.3 and MEL202 cells led to the increased phosphorylation of YAP at S127 and S397, respectively. Conversely, the ectopic expression of YAPer-ORF resulted in the pronounced dephosphorylation of endogenous YAP in OMM2.3 cells (Fig. 3h, i). Interestingly, the manipulation of YAPer-ORF in MEL290 cells did not notably alter the phosphorylation status of YAP. TRULI is a widespread agonist of YAP activity that consistently activates YAP by inhibiting its phosphorylation [33]. After we treated MEL290 cells with TRULI for 24 h, the phosphorylation levels of the YAP S127 and S397 sites substantially decreased. Moreover, after endogenously knocking down YAPer-ORF expression, this inhibitory effect of TRULI was further enhanced, which indicated that YAPer-ORF could activate YAP activity (Fig. 3j). We next examined the effects of YAPer-ORF on YAP activity in vivo, and the results indicated that YAP phosphorylation but not total YAP levels were markedly decreased in the cardiac tissues of YAPer-ORF-overexpressing mice (Fig. 3k and Supplementary Fig. 3c, d), a finding that was consistent with the in vitro results. These results demonstrated that YAPer-ORF represses YAP phosphorylation to activate YAP activity, which is more prominent in UM cells harboring the GNAQ/11 mutation or upon the activation of YAP activity.

### YAPer-ORF competes with PRP4K to dephosphorylate and sequester YAP in the nucleus

**Fig. 4:**
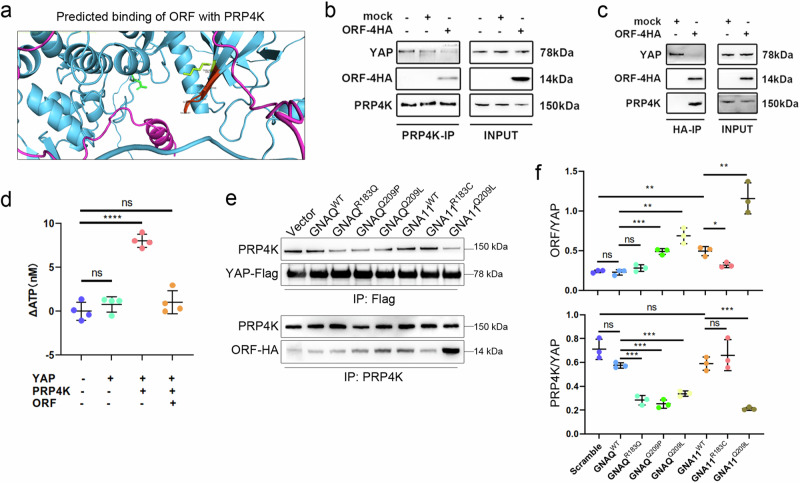
YAPer-ORF competes with PRP4K to dephosphorylate and sequester YAP in the nucleus. **a** Structural comparison of YAPer-ORF-PRP4K via AlphaFold2. **b** Coimmunoprecipitation (co-IP) assays showing the interactions of PRP4K with endogenous YAP and ectopic ORF proteins. **c** Co-IP assays showing interactions of ectopic ORF with endogenous YAP and PRP4K proteins. **d** An in vitro kinase assay was used to assess the kinase activity of PRP4K with/without YAPer-ORF. Co-IP assays (**e**) and quantification (**f**) showing interactions of ectopic YAP with endogenous PRP4K proteins (upper); Co-IP assays showing interactions of endogenous PRP4K with ectopic ORF proteins (lower).

The precise regulatory mechanisms of YAP in the nucleus remain poorly understood, despite the nuclear localization of YAPer-ORF. Recently, the nuclear kinase PRP4K was reported to specifically catalyze YAP phosphorylation to regulate intranuclear YAP release [22]. Therefore, we investigated whether there was an interaction between YAPer-ORF and PRP4K. A structural comparison of YAPer-ORF and PRP4K via AlphaFold2 predicted possible overlapping binding sites in the protein kinase domains of PRP4K and YAP (Fig. 4a). Co-IP analysis demonstrated a direct interaction between YAPer-ORF and PRP4K (Fig. 4b, c). Our in vitro kinase activity assay demonstrated that the kinase activity of Myc-PRP4K was almost completely suppressed by its interaction with YAPer-ORF (Fig. 4d**)**. Our co-IP results also revealed that the knockdown of endogenous GNAQ^wt^ and GNA11^wt^ attenuated the PRP4K-YAP interaction and that mutations R183Q, Q209P, and Q209L in GNAQ, as well as the Q209L mutation in GNA11, abolished the binding between PRP4K and YAP (Fig. 4e, f). Consistently, we observed that the Q209P and Q209L mutations in GNAQ enhanced the interaction between YAPer-ORF and PRP4K (Fig. 4e, f). In conclusion, these results indicate that YAPer-ORF competitively binds with PRP4K to block YAP phosphorylation, whereas mutations in GNAQ promote the interaction of YAPer-ORF and PRP4K and abolish the interaction between YAP and PRP4K. Therefore, the level of phosphorylated YAP within the nucleus was synergistically decreased, which led to an extremely high level of activated YAP signaling.

### YAPer-ORF facilitates the nuclear translocation of the GNAQ/11 mutant to suppress PRP4K transcription

**Fig. 5:**
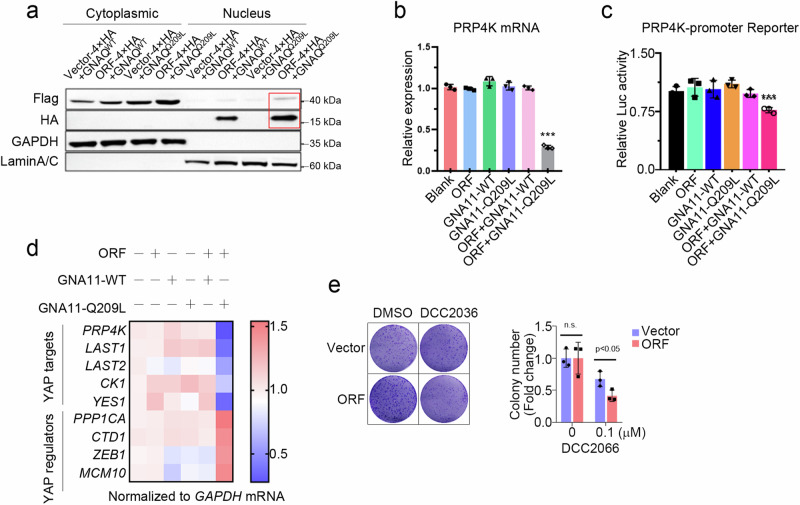
YAPer-ORF facilitates the nuclear translocation of mutant GNAQ/11 to suppress PRP4K transcription. **a** YAPer-ORF facilitates the nuclear translocation of mutant GNAQ/11. ORF-4×HA fusion constructs and GNAQ/11 mutants with 3×Flag fusion constructs were cotransfected into HEK-293T cells for 24 h, and nuclear and cytoplasmic proteins were assessed by western blotting with anti-HA and anti-Flag antibodies, respectively. GAPDH and Lamin A/C are markers of the cytoplasm and nucleus. **b** Intranuclear GNAQ/11Q209L significantly decreased PRP4K mRNA levels. Blank: empty vector; ORF: only ORF overexpression; GNA11-WT: only wild-type GNA11 protein overexpression; GNA11-Q209L: mutant GNA11 protein overexpression; ORF + GNA11-WT: intranuclear wild-type GNA11 protein overexpression; ORF + GNA11-Q209L: intranuclear wild-type GNA11 protein overexpression. **c** Intranuclear GNAQ/11 Q209L significantly decreased PRP4K transcription activity. **d** Intranuclear GNAQ/11 Q209L activated YAP signaling activity. The indicated genes were YAP signaling target genes. **e** Colony formation of control or ORF-overexpressing 92.1 cells with DCC2066. The data in **b** and **c** are presented as means ± SDs. The error bars represent the SDs of triplicate experiments. All *p* values were calculated using two-tailed unpaired Student’s *t* tests. ***p < 0.001.

On the basis of the above findings, YAPer-ORF is localized in the nucleus, and YAPer-ORF interacts specifically with GNAQ/11. To test whether cytoplasmic GNAQ/11 can be coerced into the nucleus by YAPer-ORF, we designed the following five sets of constructs: empty and GNAQ/11 wild-type, YAPer-ORF and GNAQ/11 wild-type, empty and GNAQ/11 Q209L mutant, and YAPer-ORF and GNAQ/11 Q209L mutant. We cotransfected each of the above five sets of constructs into HEK-293 cells and assessed the nucleoplasmic protein profiles. We found that YAPer-ORF better facilitated the translocation of the GNAQ/11 Q209L mutant into the nucleus (Fig. 5a). To explore the function of the GNAQ/11 mutant in the nucleus, we first examined the transcription levels of critical regulators in the YAP signaling pathway, and the results showed that the GNAQ/11 mutant in the nucleus significantly downregulated partial gene expression to activate YAP signaling (Fig. 5d). We used PRP4K as an example to explore the molecular mechanism underlying the decrease in RP4K transcript levels induced by the GNAQ/11 mutant. We found that the ability of GNAQ/11 to bind different lengths of the promoter region of PRP4K was not the same (Fig. 5b, c), which may imply that GNAQ/11 is responsible for the modulation of the triplex genome structure in the promoter region of target genes. Additionally, we discovered that DCC2036, an agonist of PRP4K, attenuated the promotion of proliferation mediated by YAPer-ORF (Fig. 5e). The above results consistently support that YAPer-ORF triggers abnormally increased YAP signaling activity through the comprehensive inhibition of PRP4K.

### Neutralizing YAPer-ORF inhibits the growth of GNAQ/11 mutant UM tumors

**Fig. 6:**
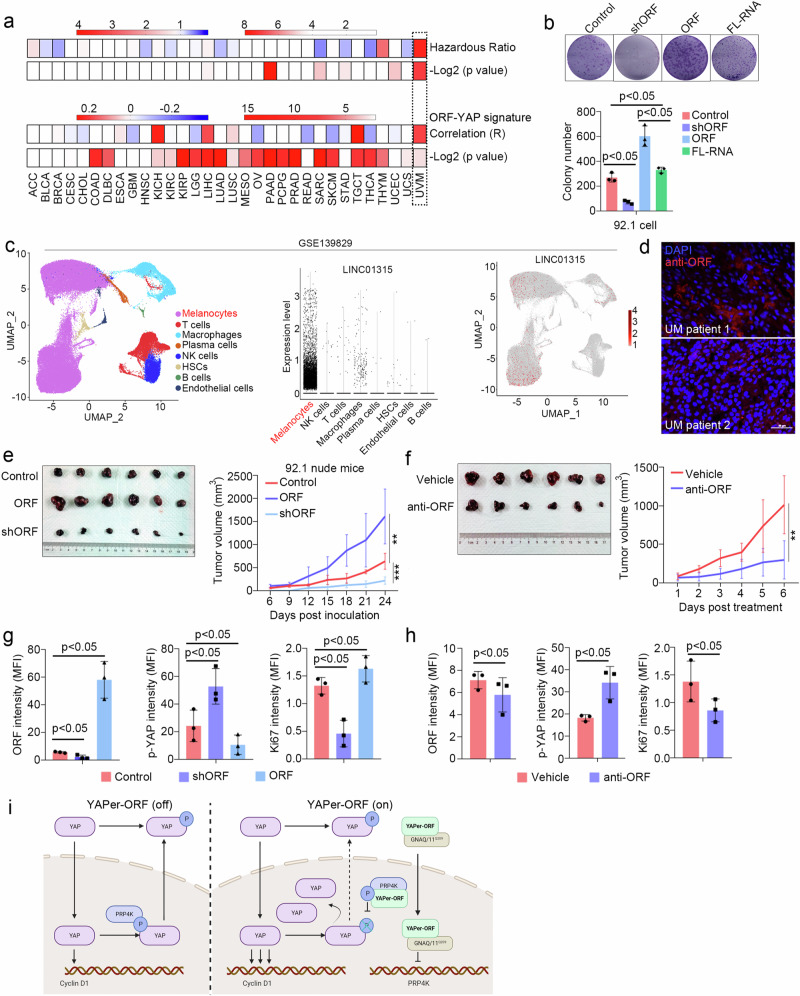
Neutralizing YAPer-ORF inhibits the growth of GNAQ/11-mutant UM tumors. **a** Correlation between YAPer-ORF and cancers from the TCGA database. **b** Colony formation assay and quantification of 92.1 cells with ORF knockdown, overexpression or full-length expression. **c** Expression of LINC01315 in different cell types from the scRNA-seq analysis of the GSE139829 dataset. **d** Pathogenic sections of UM tumors from patients were immunostained with an anti-ORF antibody. **e**–**h** Nude mice were subcutaneously injected with 92.1 cells with/without treatment. Images and tumor growth curves (**e**) for 92.1 cells with ORF knockdown or overexpression and the expression of YAPer-ORF, p-YAP and Ki67 was assessed via immunofluorescence staining of pathological sections (**f**). Images and growth curves for WT 92.1 cells intratumorally injected with and anti-ORF antibody (**g**), and the quantification of the expression of ORF, p-YAP and Ki67 in corresponding sections (**h**). **i** Schematic diagram of the role of YAPer-ORF in the nuclear activity of YAP.

We then examined the survival association of LINC01315 across human cancers by analyzing data from the TCGA database. In this analysis, UM presented the highest hazard ratio (HR) and a highly significant correlation between the ORF and the YAP signature (Fig. 6a). Colony formation assays revealed the significant promotion of tumor growth upon YAPer-ORF overexpression (Fig. 6b). Furthermore, the scRNA-seq data from GSE139829 indicated that LINC01315 was predominantly expressed in melanoma cells (Fig. 6c). Consistently, UM patient samples presented high levels of YAPer-ORF expression, as shown by immunofluorescence (IHC) staining (Fig. 6d and Supplementary Fig. 4a).

To determine whether YAP is the central factor mediated by the regulatory role of YAPer-ORF in tumorigenesis in vivo, we conducted xenograft studies involving the overexpression and knockdown of YAPer-ORF in the 92.1 (Q209L) cell line. The results demonstrated that YAPer-ORF overexpression promoted the growth of 92.1 xenografts (Fig. 6e). Considering the important role of YAP activation in maintaining cell proliferation and UM survival, the overexpression of YAPer-ORF led to a decrease in phosphorylated YAP (p-YAP) and facilitated tumor growth in vivo (Fig. 6g). Conversely, YAPer-ORF knockdown significantly reduced the tumor burden and concurrently resulted in p-YAP accumulation (Fig. 6e, g). Consistent with the earlier in vitro findings (Fig. 3), these data indicate that YAPer-ORF promotes UVM cell growth through the nuclear accumulation of YAP in vivo.

To test the therapeutic potential of YAPer-ORF inhibition, we developed in-house monoclonal anti-YAPer-ORF neutralizing antibodies. The intratumoral injection of anti-YAPer-ORF substantially suppressed tumor growth (Fig. 6f). Furthermore, IHC analysis of tumor tissue sections revealed that the anti-YAPer-ORF antibody decreased the level of YAPer-ORF and the percentage of Ki67-positive cells while increasing the intensity of p-YAP in vivo (Fig. 6h and Supplementary Fig. 4b). These findings provide further validation that YAPer-ORF regulates tumor growth by influencing YAP phosphorylation.

## Discussion

The YAP signaling pathway is known to play essential roles in organ development, tissue regeneration, and tumorigenesis [1–3]. *LINC01315* has been implicated in the pathogenesis of multiple cancers, including thyroid, breast, and gastric [34–37]. Li et al., conducted functional studies on LINC01315 in exosomes from colorectal cancer stem cells, demonstrating that LINC01315 enhances cell viability, proliferation, stemness, and migration in colorectal cancer [38, 39]. However, these findings may be indicative of LINC01315’s RNA-mediated effects rather than its protein-coding functions. In this study, we revealed a novel mechanism by which YAPer-ORF functions as a critical regulator of YAP signaling at multiple levels. First, YAPer-ORF competitively binds to the YAP-specific nuclear kinase PRP4K, promoting the nuclear retention of YAP by decreasing its phosphorylation so that the transcription of YAP-activated downstream target genes, such as CCND1, an essential regulator of the cell cycle, is enhanced, which inevitably stimulates the cell cycle and growth. Second, we observed an interaction between YAPer-ORF and the G protein subunit GNAQ/11, a key component of the YAP signaling pathway that is typically membrane-bound. Surprisingly, YAPer-ORF facilitates the translocation of GNAQ/11 into the nucleus, where it impedes the YAP phosphorylation mediated by PRP4K, resulting in further activation of YAP signaling. Third, these phenomena were particularly pronounced in the context of GNAQ/11 mutations. UM represents a well-established model of GNAQ/11 mutation, as more than 95% of UM patients harbor pathogenic mutations in GNAQ/11 [19, 40]. Consequently, UM is often associated with aberrantly amplified YAP signaling. Notably, we found that, compared with wild-type GNAQ/11, YAPer-ORF preferentially binds to the Q209L mutant of GNAQ/11, significantly enhancing the nuclear translocation of Q209L. This further strengthens the impact of YAPer-ORF on PRP4K-mediated YAP signaling extension in UM because only the entry of the Q209L mutant of GNAQ/11 exclusively inhibited PRP4K at the transcription level, which is equal to a second hit. Overall, our study reveals a previously unrecognized mechanism underlying the abnormal activation of YAP signaling in UM, providing novel mechanistic insights into the regulatory functions of YAPer-ORF in YAP signaling (Fig. 6i).

Our findings have significant therapeutic implications, highlighting YAPer-ORF as a promising target for the treatment of diseases characterized by aberrant YAP activity. Importantly, we demonstrated that the administration of an anti-YAPer-ORF-specific neutralizing antibody effectively inhibited YAP activity and suppressed tumor growth mediated by YAPer-ORF in an animal model. Moreover, our scRNA-seq analysis revealed that LINC01315 was exclusively expressed in UM tumors, emphasizing its potential as a specific therapeutic target. Furthermore, a pancancer analysis revealed positive associations of LINC01315 expression with shortened patient survival and increased YAP activity. These results underscore the clinical relevance of YAPer-ORF encoded by LINC01315. However, it is essential to acknowledge the limitations of our study. Further evaluation is necessary to assess the relatively short half-life and potentially low affinity of the anti-YAPer-ORF neutralizing antibody before considering its clinical application [41, 42].

This study also has other limitations. We identified a G-quadruplex (G4) structure in the 5’ UTR sequence of LINC01315 that has the capacity to regulate the translation of the lncRNA-encoded small protein [43]. Nevertheless, the in vivo mechanisms governing the precise modulation of YAPer-ORF translation have yet to be fully elucidated.

## Supplementary information

Supplementary information

Original western blots

## Data Availability

All the raw RNA-seq data generated from this study have been uploaded to the NCBI Gene Expression Omnibus (GEO), and the accession number is GSE272013.
