# Supplementary material for: The LINC01315-encoded small protein YAPer-ORF competes with PRP4k to hijack YAP signaling to aberrantly promote cell growth
Source: Cell Death Differ. 2025 Feb 17;32(8):1428–40. doi: 10.1038/s41418-025-01449-z (PMC12325761; doi:10.1038/s41418-025-01449-z)

**Supplementary information**

**Supplementary Fig. 1: Validation of the specificity of an in-house antibody against YAPer-ORF**

**a** Fluorescence of candidate monoclonal antibodies (12D2, 14F3, 3C4, 4B1, and 9E4) in different stably transformed cell lines (MEL290 control, MEL290 ORF-overexpressing, MEL290 shORF, and 92.1 cells). **b** Western blot results of the monoclonal antibody detection against endogenous expression of YAPer-ORF in OMM2.3 cells.

**Supplementary Fig. 2: Proliferation and apoptosis assay in cells with knockdown or overexpression of YAPer-ORF**

**a** Altering YAPer-ORF expression levels significantly affected proliferation but not apoptosis in normal epithelial cells (ARPE-19) or multiple UM tumor cell lines (OMM2.3, Mel290, and Mel202 cells). **b** The heatmap represents the percentage of apoptotic cells in the field of view for six experiments. All p values were calculated using two-tailed unpaired Student’s t tests. n. s. represents no statistical significance.

**Supplementary Fig. 3: YAPer-ORF did not change the size of heart cells and total YAP expression**

**a** Cardiomyocyte volume as determined by fluorescence; cardiomyocyte membranes (WAG). **b** histograms of the results for each visual field for six experiments. **c** Total YAP expression levels in the cardiac tissues of YAPer-ORF-overexpressing and control mice. **d** histograms of the results for each visual field for six experiments.

**Supplementary Fig. 4: IHC staining of YAPer-ORF, p-YAP and Ki67 in 92.1 tumors with YAPer-ORF knockdown or overexpression and YAPer-ORF staining in UM patient samples.**

**a** YAPer-ORF staining results in UM patient adjacent tissues. **b** Immunohistochemistry revealed that YAP activity (p-YAP) and proliferation (Ki67) varied substantially in nude mouse tumors formed from different stably transformed strains of UM cells (92.1-Control, 92.1-shORF, 92.1-ORF, and 92.1-Vehicle).


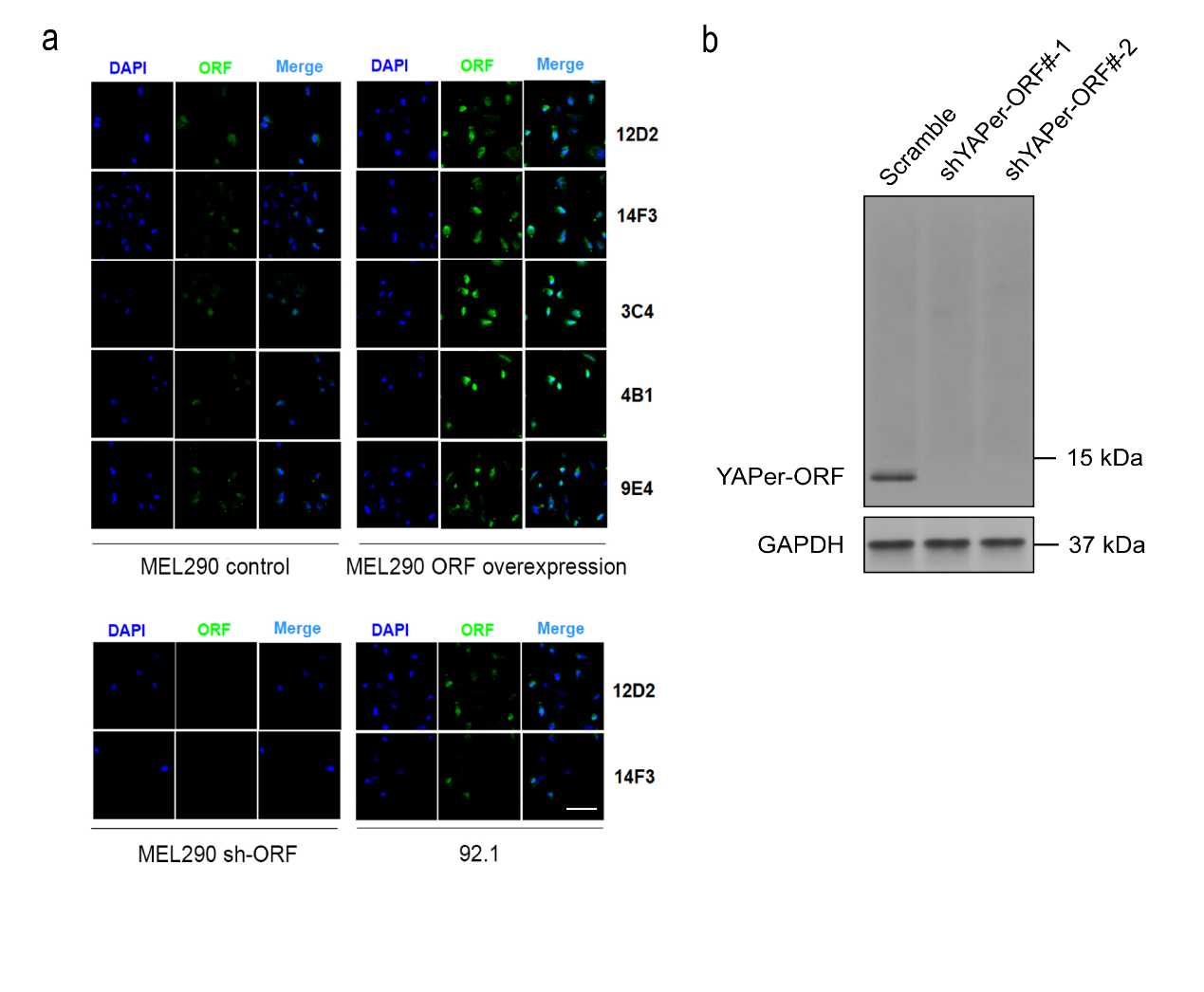


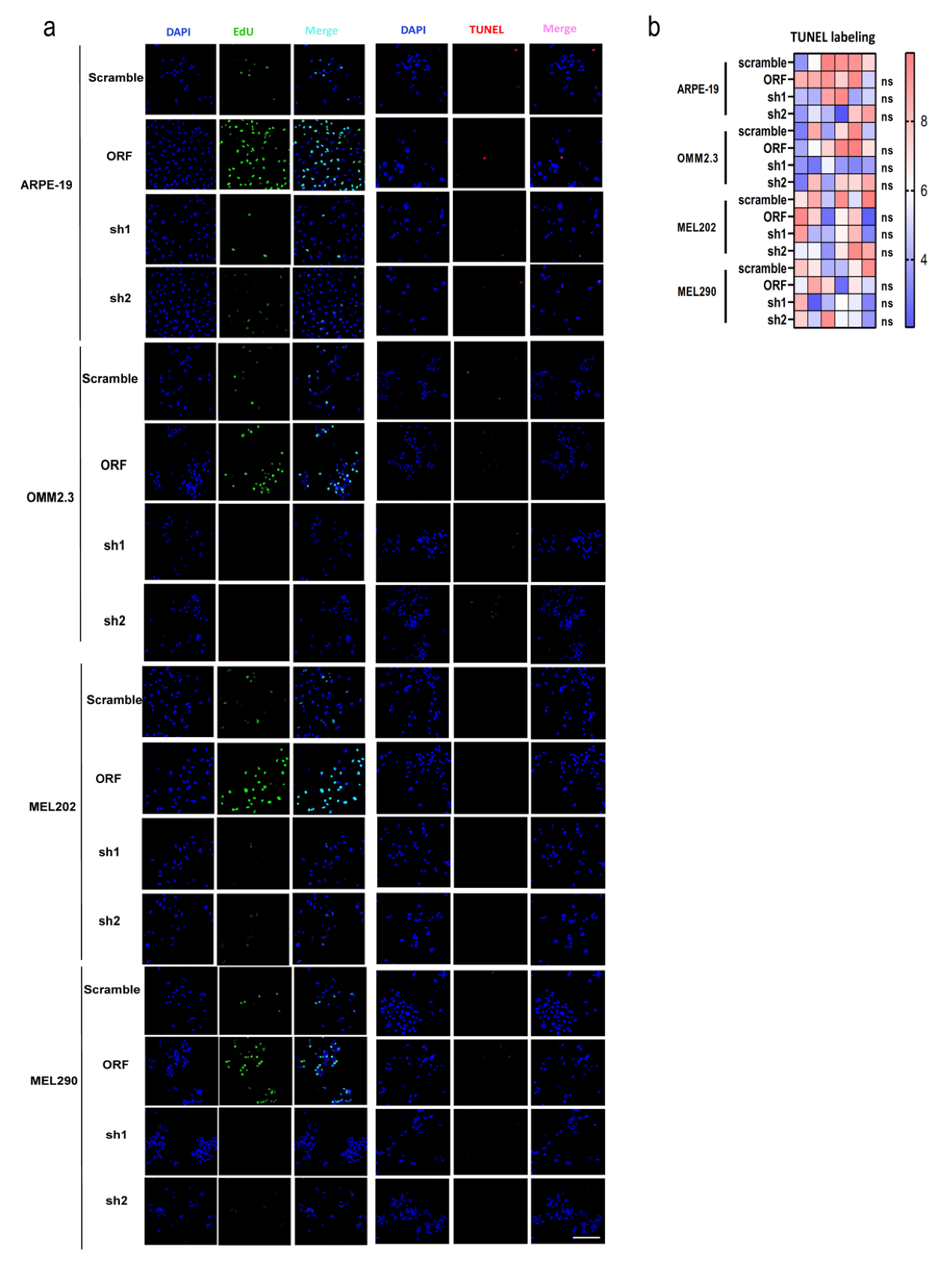


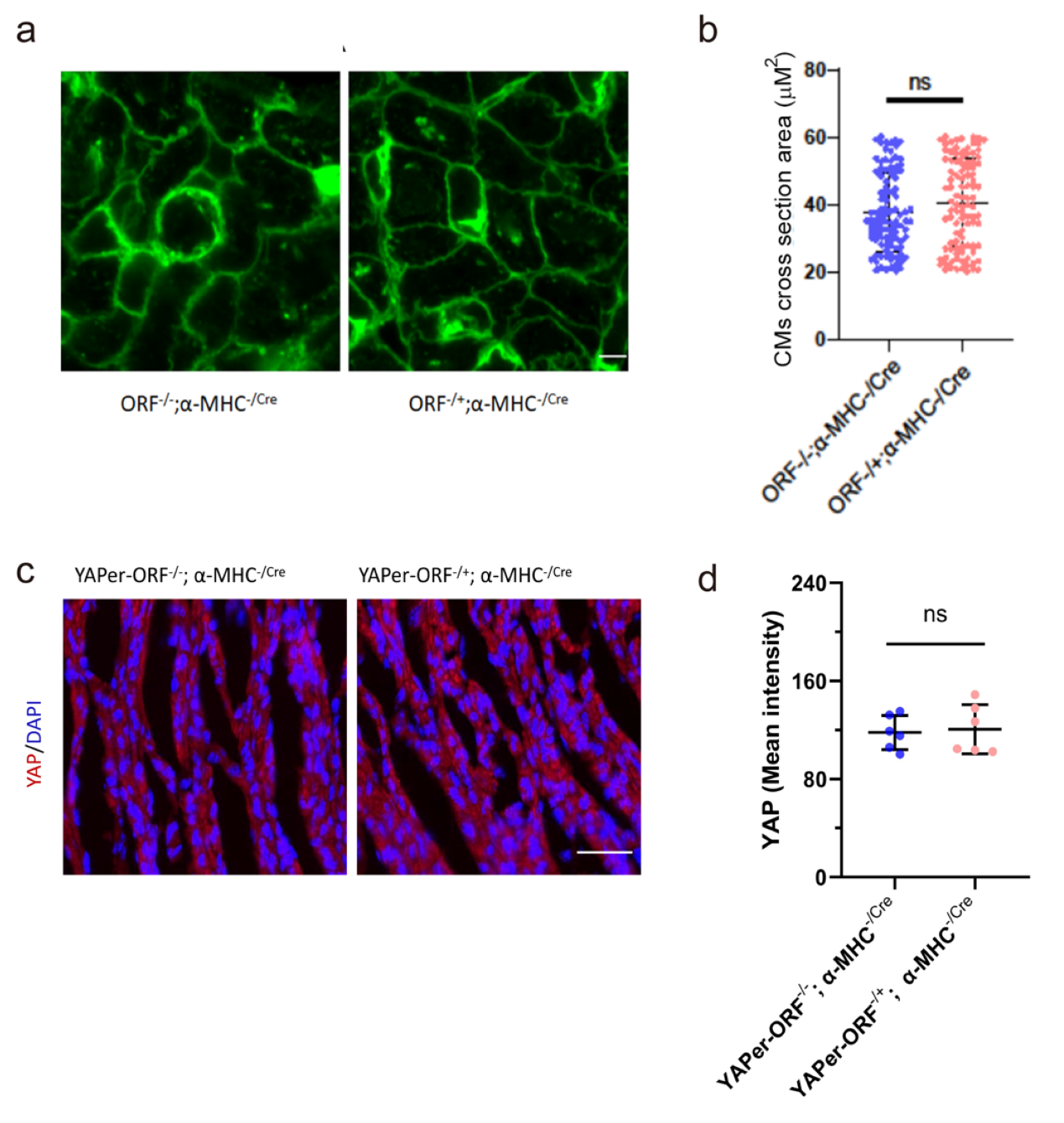


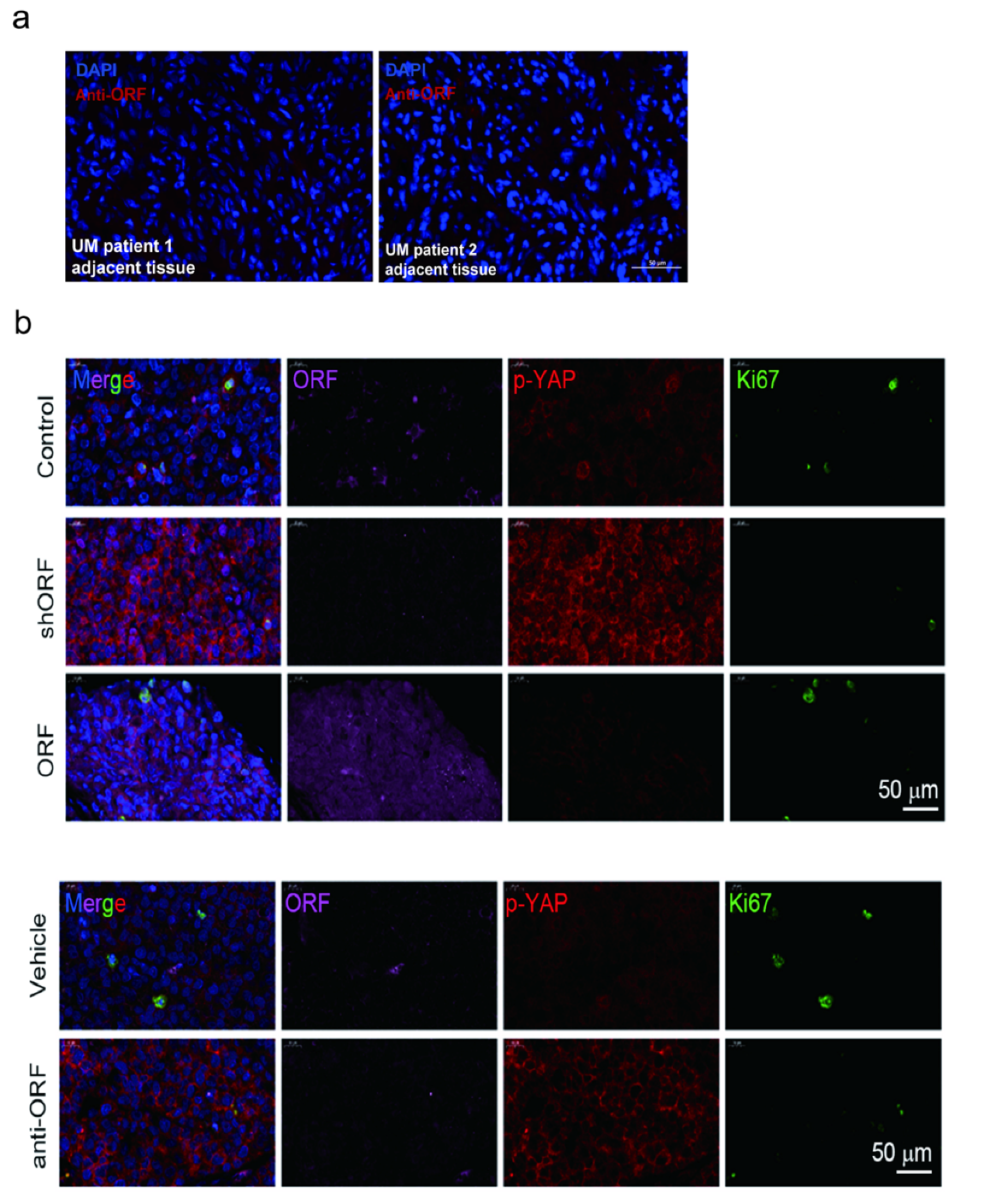

Supplement: Supplementary file 1 — Supplementary information [file 41418_2025_1449_MOESM1_ESM.docx]
