# Supplementary material for: The LINC01315-encoded small protein YAPer-ORF competes with PRP4k to hijack YAP signaling to aberrantly promote cell growth
Source: Cell Death Differ. 2025 Feb 17;32(8):1428–40. doi: 10.1038/s41418-025-01449-z (PMC12325761; doi:10.1038/s41418-025-01449-z)

Fig. 1c

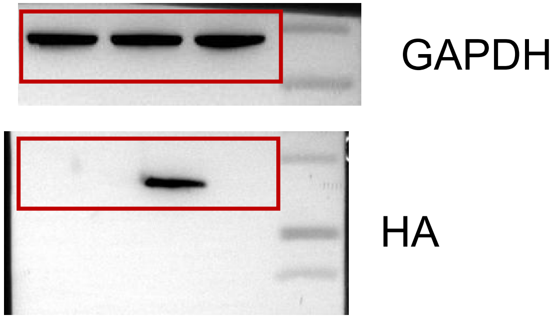

Fig.1f

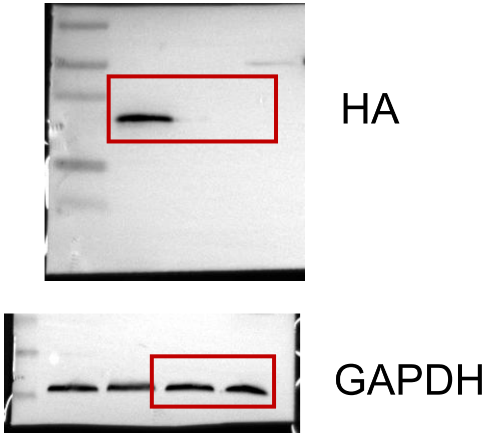

Fig.1e

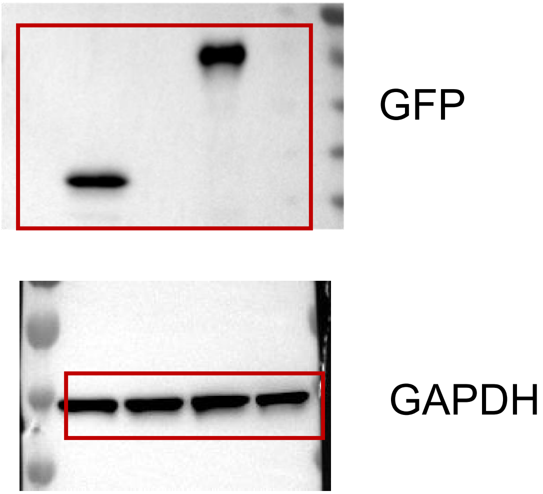

Fig. 1g

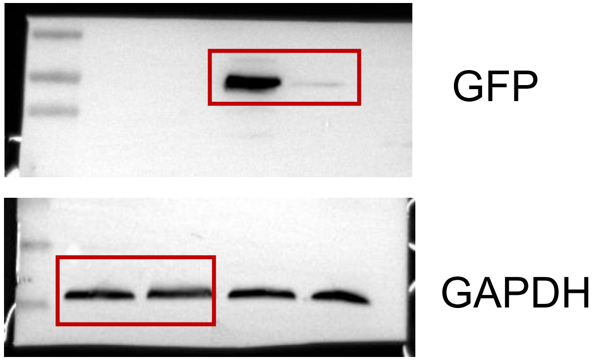

Fig. 1i

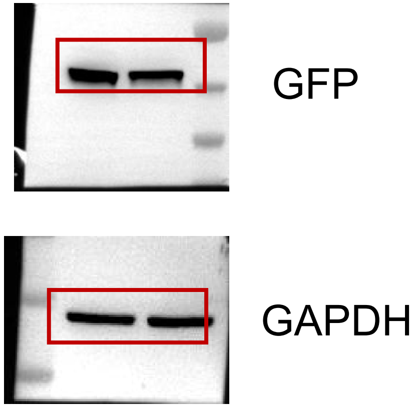

Fig. 1o

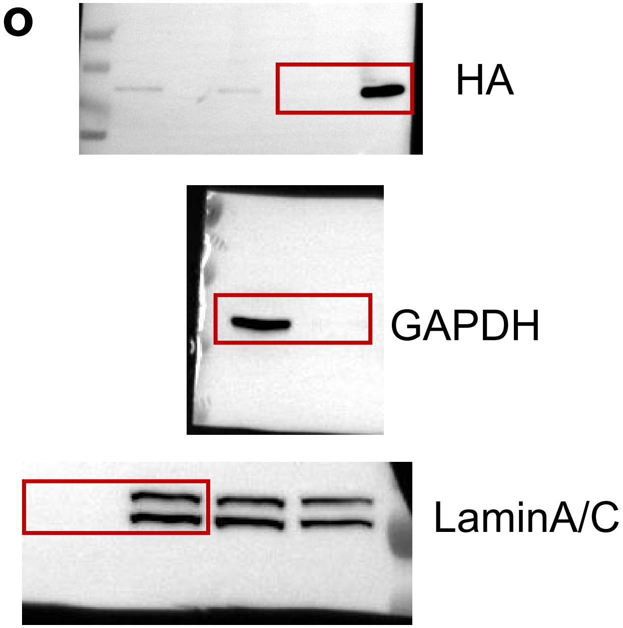

Fig. 1p

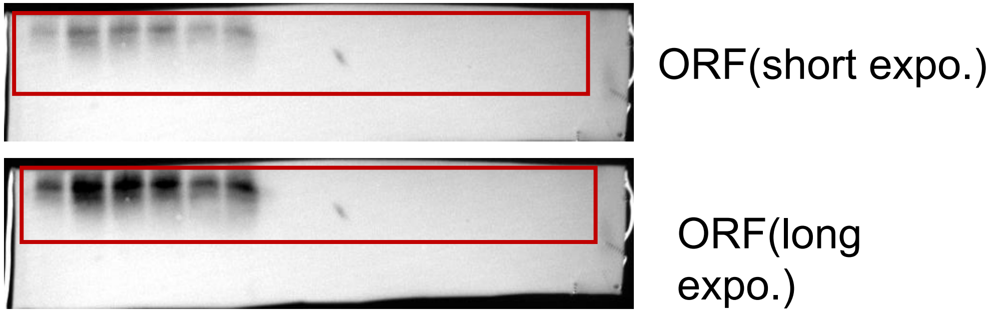

Fig. 2g

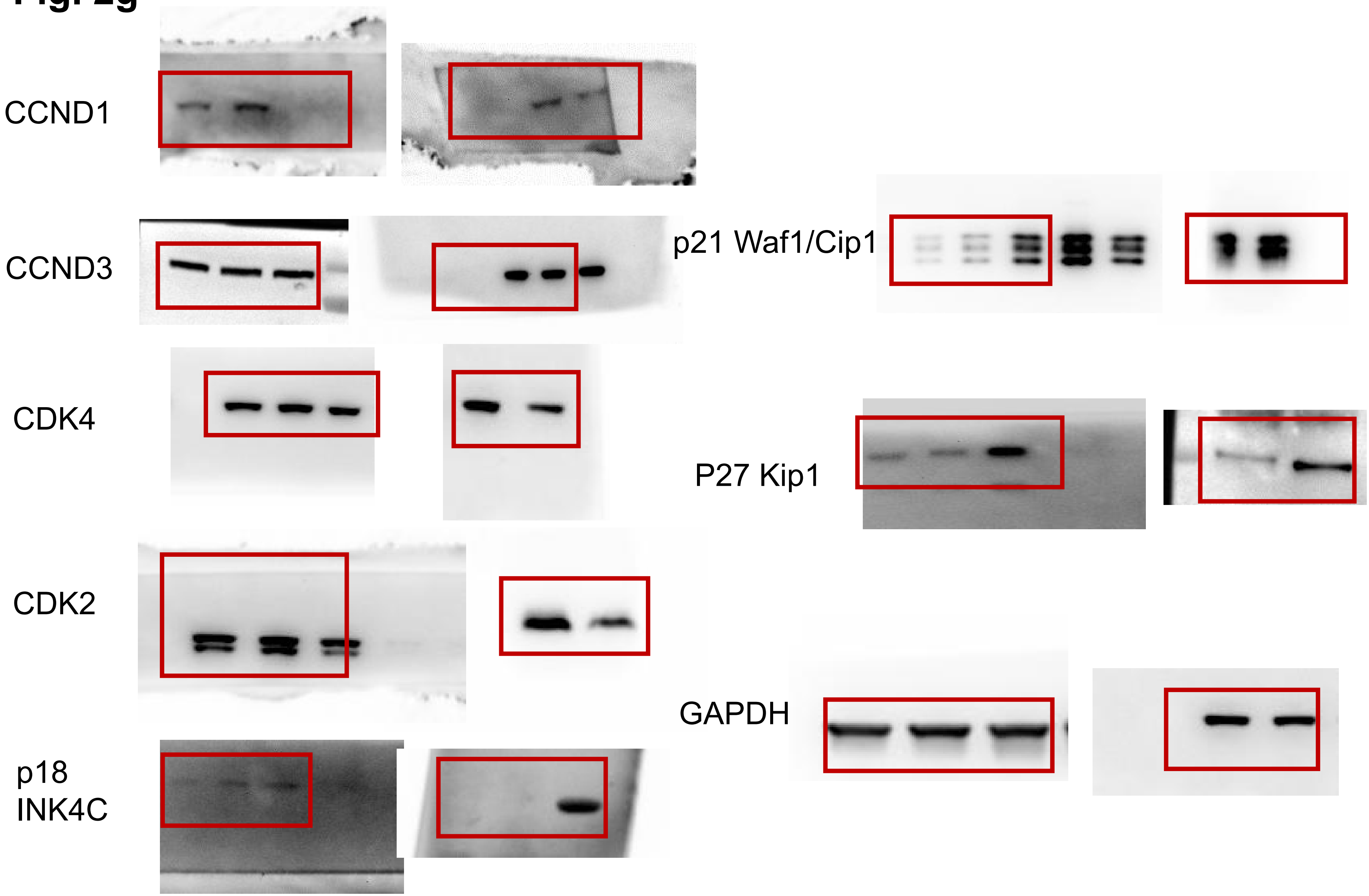

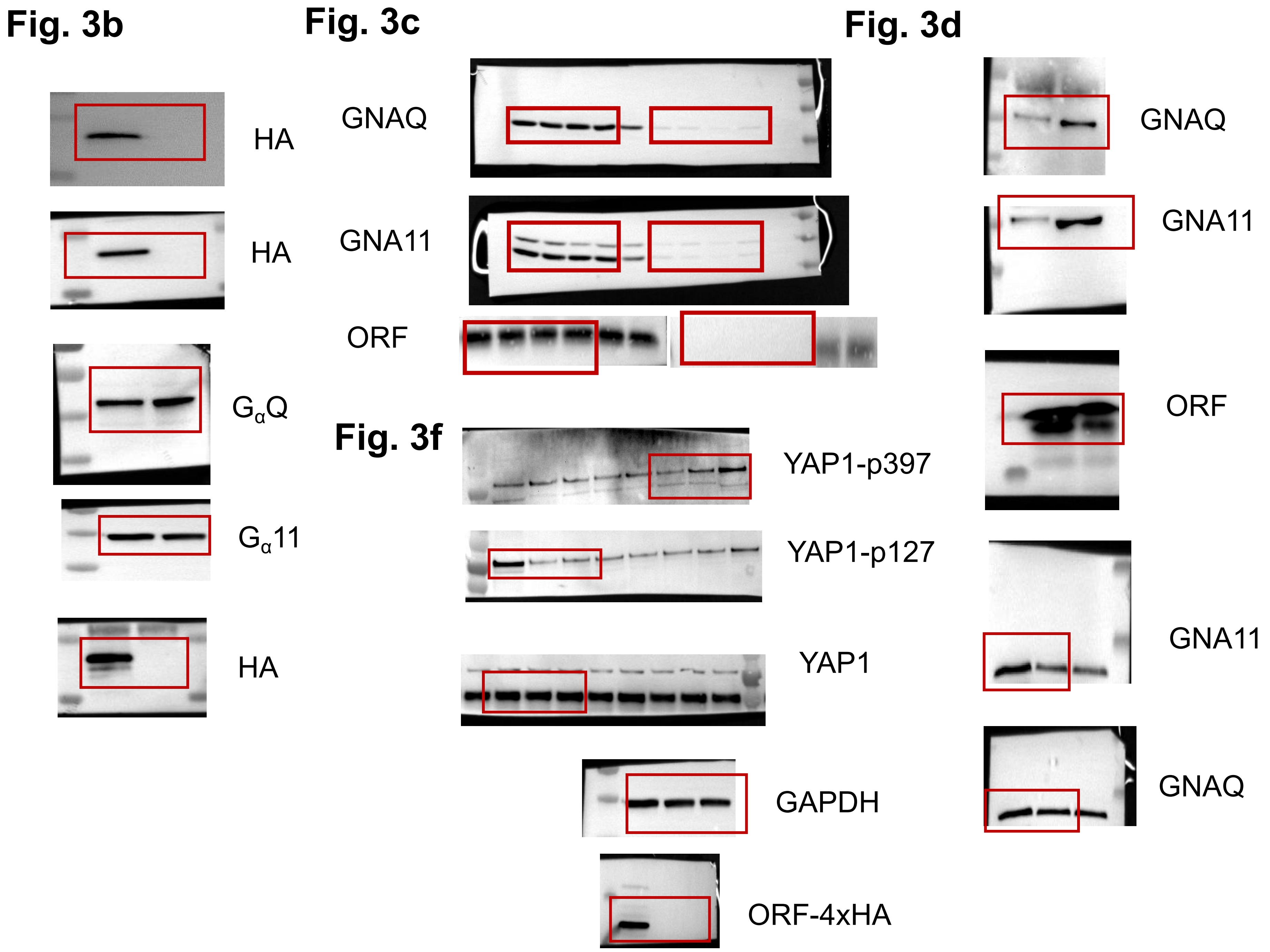
Fig. 3b
HA
HA
$G_{\alpha}Q$
$G_{\alpha}11$
HA
Fig. 3c
GNAQ
GNA11
ORF
Fig. 3f
YAP1-p397
YAP1-p127
YAP1
GAPDH
ORF-4xHA
Fig. 3d
GNAQ
GNA11
ORF
GNA11
GNAQ

Fig. 3h

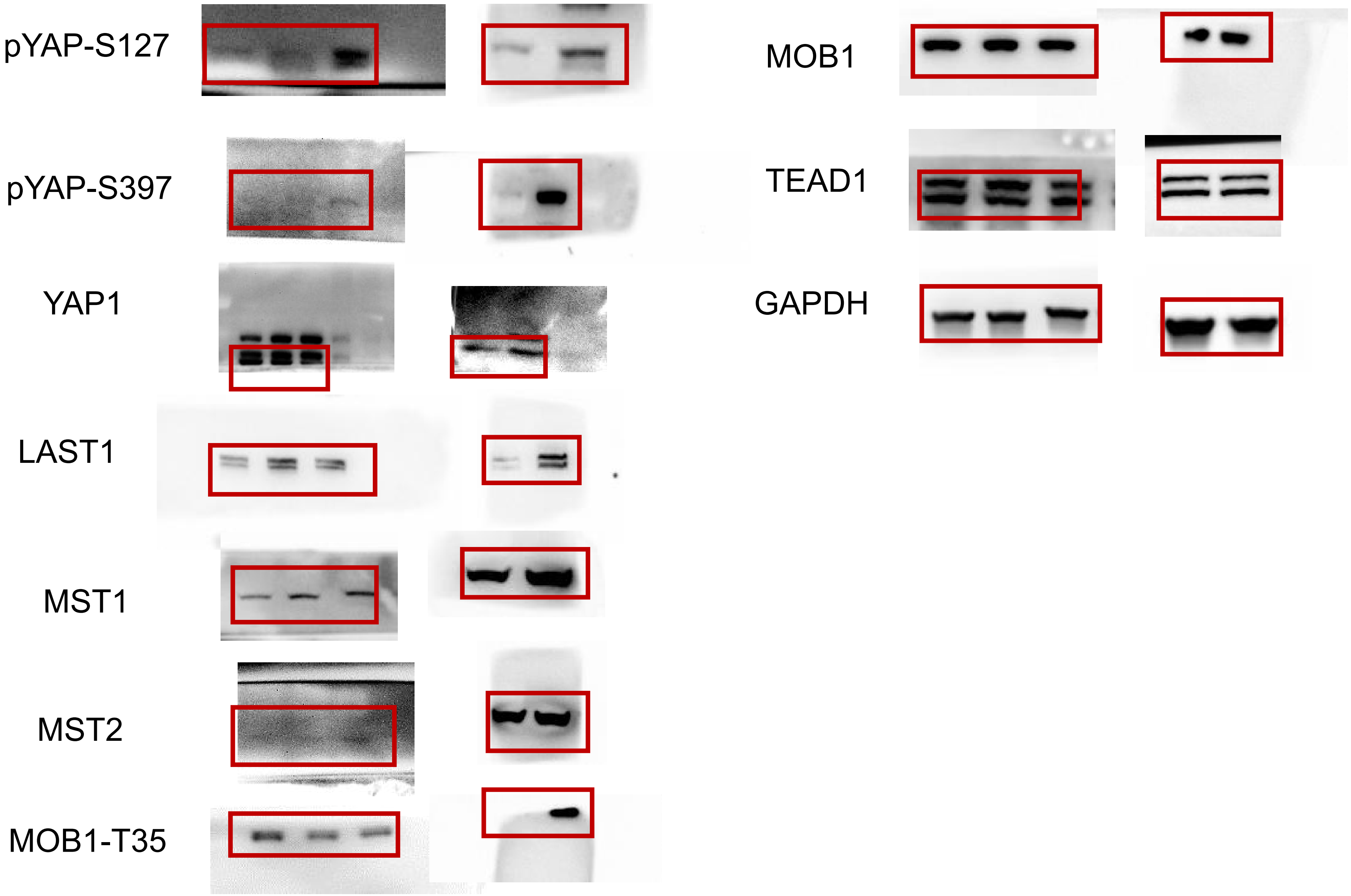
pYAP-S127
pYAP-S397
YAP1
LAST1
MST1
MST2
MOB1-T35
MOB1
TEAD1
GAPDH

Fig. 3j

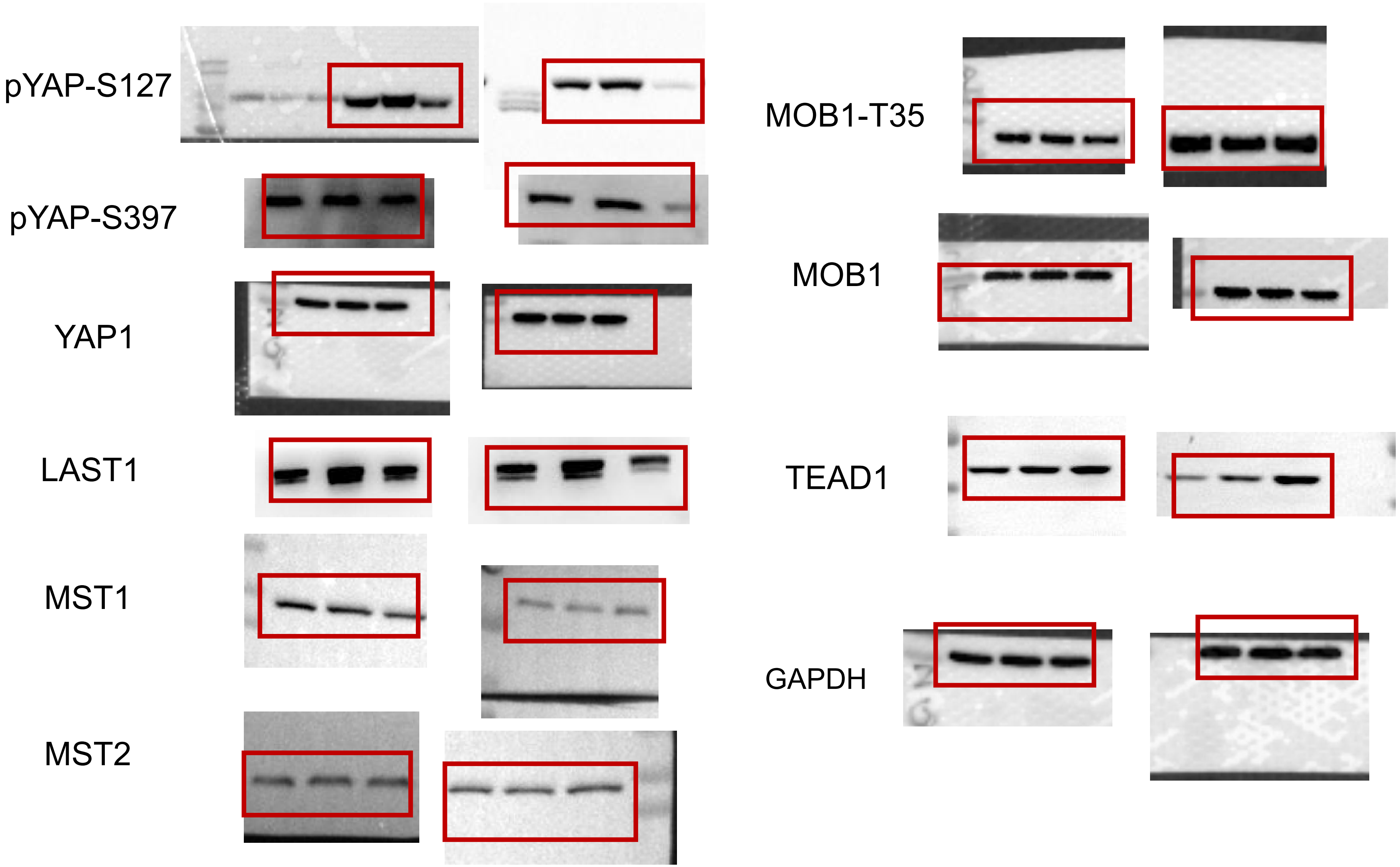
pYAP-S127
pYAP-S397
YAP1
LAST1
MST1
MST2
MOB1-T35
MOB1
TEAD1
GAPDH

Fig. 4b

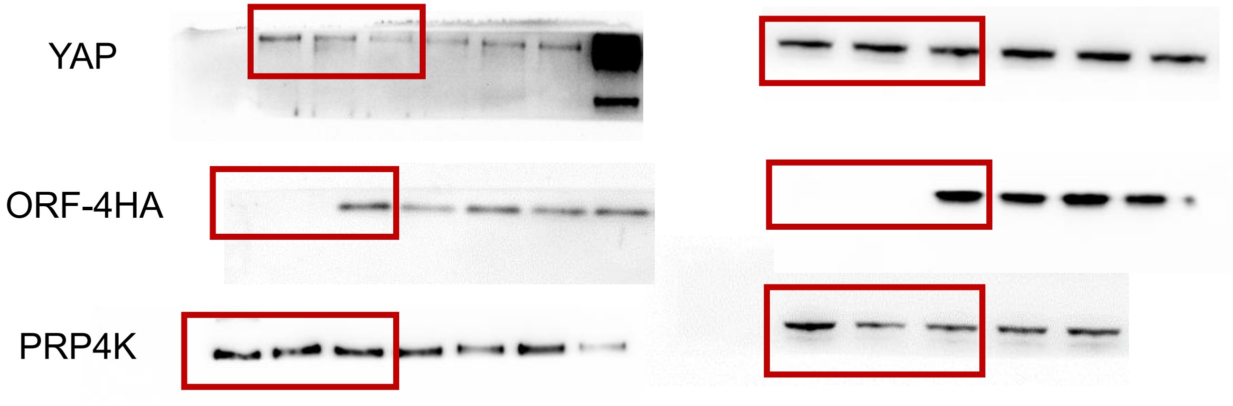

Fig. 4c

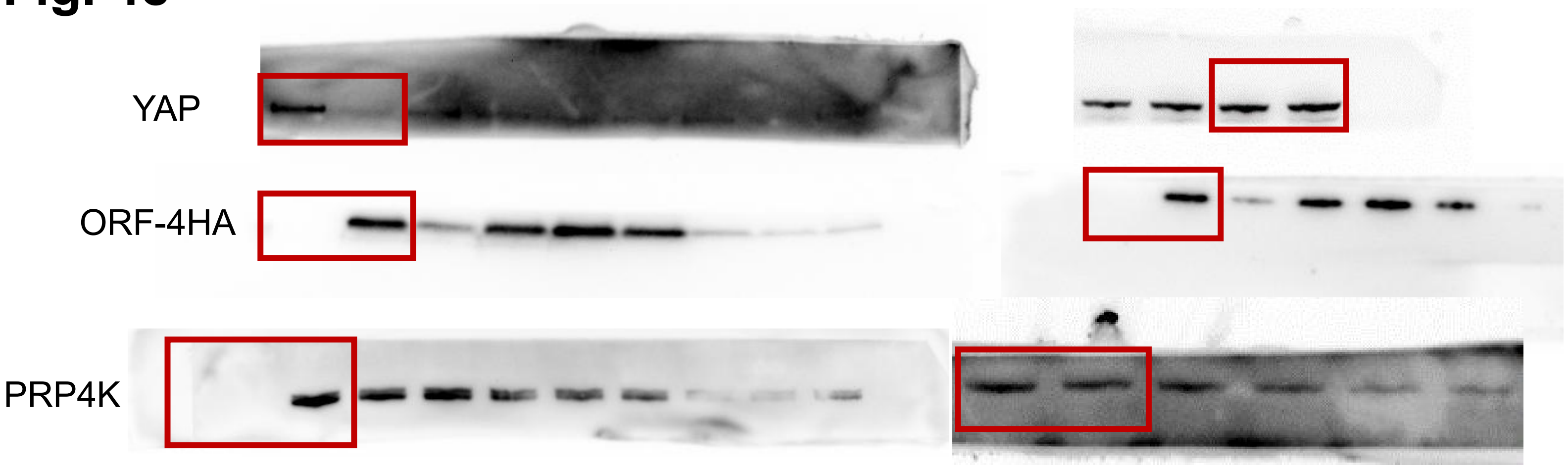

Fig. 4e

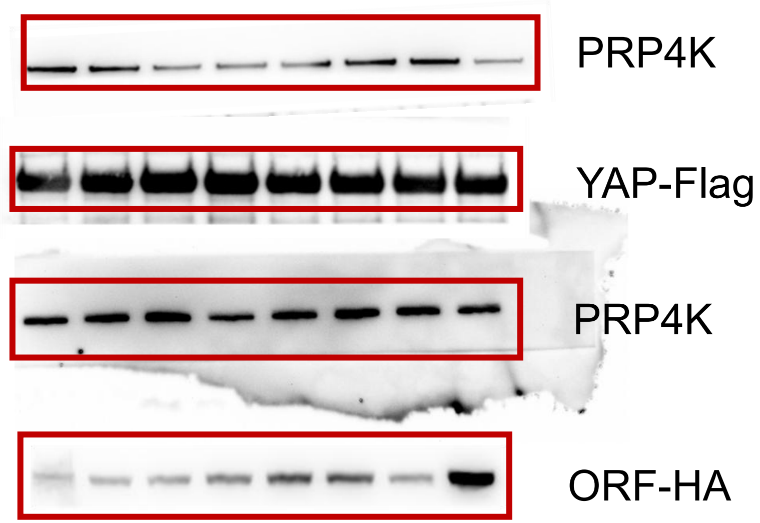

Fig. 5a

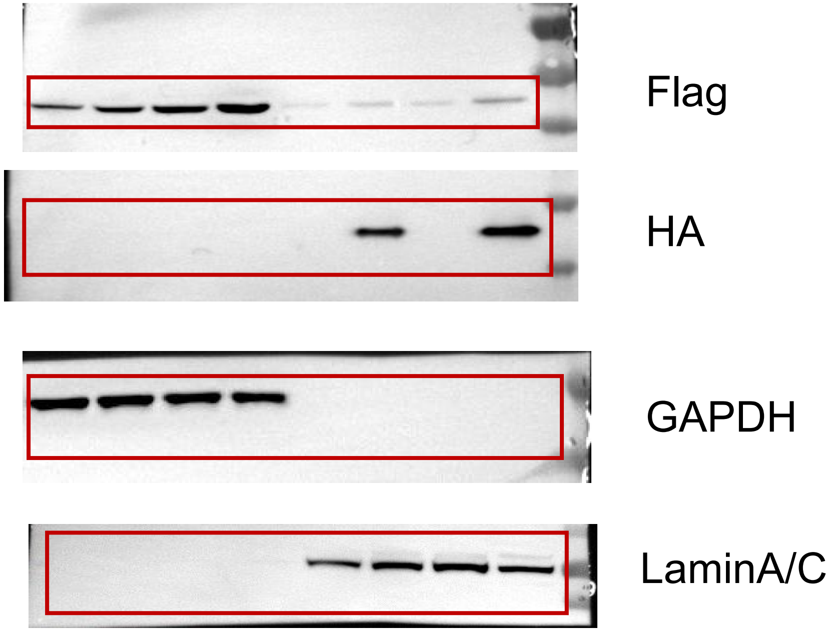

Fig. S1b

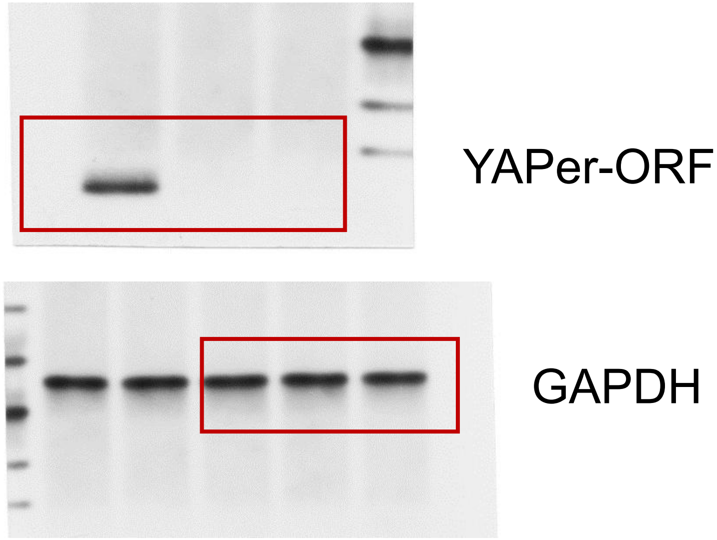

Supplement: Supplementary file 2 — Original western blots [file 41418_2025_1449_MOESM2_ESM.pdf]
